# Tendon tissue engineering: An overview of biologics to promote tendon healing and repair

**DOI:** 10.1177/20417314231196275

**Published:** 2023-09-13

**Authors:** Vera Citro, Marta Clerici, Aldo R. Boccaccini, Giovanna Della Porta, Nicola Maffulli, Nicholas R. Forsyth

**Affiliations:** 1 School of Pharmacy and Bioengineering, Keele University, Stoke-on-Trent, Staffordshire, UK; 2 Department of Materials Science and Engineering, Institute of Biomaterials University of Erlangen-Nuremberg, Cauerstrasse 6, Erlangen, Germany; 3 Department of Medicine, Surgery and Dentistry, University of Salerno, via S. Allende, Baronissi, Salerno, Italy; 4 Interdepartmental Centre BIONAM, University of Salerno, via Giovanni Paolo I, Fisciano, Salerno, Italy; 5 Department of Trauma and Orthopaedic Surgery, University Hospital ‘San Giovanni di Dio e Ruggi D’Aragona’, Salerno, Italy; 6 Vice Principals’ Office, University of Aberdeen, Kings College, Aberdeen, UK

**Keywords:** Tendon, tissue engineering, mesenchymal stem cells, extracellular vesicles

## Abstract

Tendons are dense connective tissues with a hierarchical polarized structure that respond to and adapt to the transmission of muscle contraction forces to the skeleton, enabling motion and maintaining posture. Tendon injuries, also known as tendinopathies, are becoming more common as populations age and participation in sports/leisure activities increases. The tendon has a poor ability to self-heal and regenerate given its intrinsic, constrained vascular supply and exposure to frequent, severe loading. There is a lack of understanding of the underlying pathophysiology, and it is not surprising that disorder-targeted medicines have only been partially effective at best. Recent tissue engineering approaches have emerged as a potential tool to drive tendon regeneration and healing. In this review, we investigated the physiochemical factors involved in tendon ontogeny and discussed their potential application in vitro to reproduce functional and self-renewing tendon tissue. We sought to understand whether stem cells are capable of forming tendons, how they can be directed towards the tenogenic lineage, and how their growth is regulated and monitored during the entire differentiation path. Finally, we showed recent developments in tendon tissue engineering, specifically the use of mesenchymal stem cells (MSCs), which can differentiate into tendon cells, as well as the potential role of extracellular vesicles (EVs) in tendon regeneration and their potential for use in accelerating the healing response after injury.

## Introduction

Tendons are well-organized, dense connective tissues that respond and adapt to the transmission of contraction forces by muscles to the skeleton, allowing motion and maintenance of posture.^1^ Tendinopathies are common musculoskeletal disorders that affect a wide spectrum of society.

In tendinopathy, the pathological changes can be interpreted as a failure in the homeostatic response of the tendon^2^ to adapt to altered mechanical loading, resulting in permanent changes in the native tendon structures and mechanics.^2,3^ At least three phases of tendinopathy have been hypothesized: reactive tendinopathy, tendon disrepair (failed healing) and degenerative tendinopathy. The continuum model for the pathogenesis of tendinopathy describes load-induced tendinopathy as a continuous evolution and readaptation of the tendon structure to the applied load.^4^

In the reactive phase, short-term adaptation to the overload results in an increase in the size of the tendon, thus reducing stresses and increasing stiffness. The preliminary engagement of ground substance in this process explains the potentially temporary nature of this first adaptation. During the disrepair phase, the tendon tissue attempts to repair increasing the number of cells and consequently the production of proteins; the increase of proteins, such as proteoglycans and collagen, induces disassembling of the matrix. The terminal degenerative phase results in an irreversible stage of pathology in which the major structural and compositional changes, cell death, tissue breakdown and loss of function with the predisposition of the tendon to further injury and rupture occur.^5^ The three main actors involved in tendon degeneration are mechanical overuse applied on the matrix, neo-vascularization via exogenous cells, and endogenous cell ageing.^6^

Tendon healing is a complex coordinated event orchestrated by numerous biologically active proteins.^7^ Adult tendons have a limited natural healing capacity and often respond poorly to current treatments focused on exercise, drug delivery, and surgical procedures. The incapacity of complete healing derives from the nature of the tendon with its low cellularity, low metabolism, and limited vascularization, which hamper the synthesis of extracellular matrix (ECM)^8^ and results in scar tissue formation and fibrosis, accompanied by alterations in the biomechanical properties of the tissue.^9^

The cellular component of the tendon is limited, and with age, it tends to diminish and change in morphology, with loss of stemness markers^10^ as also seen with tendinopathy or acute trauma. Tenocytes are responsible for homeostasis and the maintenance of tendon structure and functionality. The resident stem cell population are called tendon stem cells (TSCs) and lie parallel to collagen fibrils surrounded by the extracellular matrix proteins fibromodulin and biglycan.^11^ As the tendon is a mechanosensitive tissue and extracellular matrix (ECM) remodelling is influenced by mechanical stimulation, prolonged rehabilitation is considered a valid alternative to surgery, as it offers great support and is more efficient than pharmacological therapy.^12^ Therefore, it is of great importance to identify key molecular and cellular processes involved in the progression of tendinopathies and in tendon response to them to develop effective therapeutic strategies and drive the tissue towards regeneration.^10^ Unfortunately, the structural complexity and lack of understanding of this healing mechanism have been major obstacles to the development of current surgical methods and therapies.

The current promising approach to the development of therapeutics has been influenced by key principles from a variety of fields, such as biomechanics, developmental biology, cell and growth factor therapy, and tissue engineering.^13^ It aims to reproduce a safe and successful long-term solution for full microarchitecture and biomechanical tissue recovery. These ‘biological’ treatments likely stimulate the repair and regeneration of damaged structures, limit scar tissue formation, and improve recovery and healing times. Commonly explored therapies for tendinopathies include platelet-rich plasma (PRP), stem cells/stromal cells treatments with the addition of soluble signals like growth factors, and most recently extracellular vesicles (EVs).^14^

This review will cover the literature surrounding recent advances in tendon repair. It will transversally analyse the cause-and-effect relationship among biochemical, biological and structural properties of tendon development, providing a complete insight into the aspect to consider during the process of tissue engineering. It will first provide a general overview of tendon structure and function to be able to understand the physiological processes involved in tendinopathies. Then, current approaches for tendinopathies treatment will be shown, highlighting the future promise for tissue engineering in tendon regeneration. In this article, we focus on the potential of MSCs cells to differentiate into tendon cells and on the therapeutic roles of EVs for the promotion of tendon repair.

## Tendon ontogeny

The ECM experiences limited turnover during the life span of an individual.^15^ With age, aerobic energy production and synthesis of ECM components decrease. The shift to anaerobic metabolism allows tendons to tolerate low oxygen levels, reducing the risk of ischaemia and necrosis during extended periods of stress but also resulting in a poor and slow healing capacity.

To generate functional and self-renewing tendon tissue, it is necessary to understand the normal processes of tendon development. In particular, we need to understand which stem cell populations in the body are able to form tendons, how they can be directed to do so in culture excluding any other possible differentiation, and how their growth is controlled and monitored during the whole differentiation path.

### Developmental biology of the tendon

**Figure 1.**
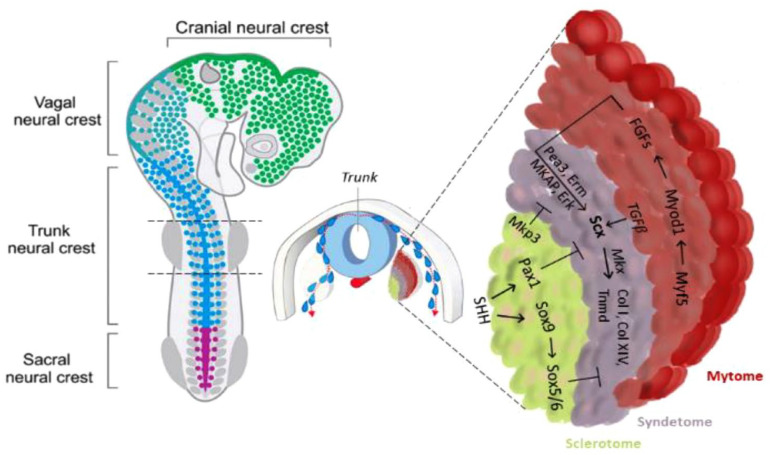
Trunk tendon differentiation model. In the axial tendon, signals from the myotome are essential for the initiation of tendon progenitor cells, whereas signals from the sclerotome, which are activated by the ventral midline sonic hedgehog signal, in turn, play an opposite role. Scx and Mkx promote axial tendon differentiation by activating extracellular matrix molecules.

Axial tendon progenitors and progenitors of the limb tendons develop differently. The vertebrate axial musculoskeletal system originates from somites: dorsally located segmental blocks of mesoderm in the embryo that lie adjacent to the natural tube and notochord.^16^ The differentiation of the tendon in the somite depends on the combination of both activating and repressing signals from its different regions. Brent et al. localized a fourth region of the somite for the first time, between the sclerotome and the dermomyotome, which locates the basic-helix-loop-helix transcription factor Scleraxis (Scx)^17^ As determined from previous studies,^17,18^ removing the dermomyotome before myotome formation or silencing the myogenic genes, such as MyoD and Myf5, which hamper muscle formation, abolish Scx expression. Therefore, myotome signals are relevant for the activation of tendon differentiation in the syndetome. Nevertheless, several fibroblast growth factors (FGFs), are expressed in the myotome resulting in the activation of the mitogen-activated protein kinase pathway, transformation-specific sequence (Ets) transcription factors, Phosphatidylinositol-4-phosphate 5-kinase (Pea3) and Ezrin/radixin/moesin (Erm), all responsible for Scx upregulation.^19^ The muscle precursor region of the somite is therefore essential for the initiation of tendon differentiation (Figure 1).

**Figure 2.**
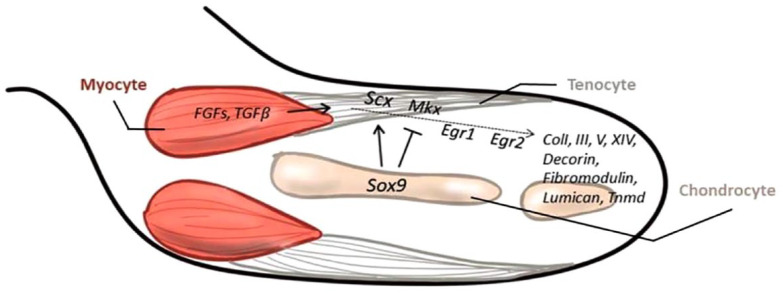
Development of limb tendon in embryogenesis. In the limb, Scx and Sox9 are the first signals for tendon progenitor cell initiation, whereas Mkx and early growth response 1 and 2 are the second signals for tendon differentiation and maturation. Sox9 is involved in the initial stage and changes to play an opposite role subsequently. Both are synergically involved in limb tendon development by interacting with related growth factors.

**Figure 3.**
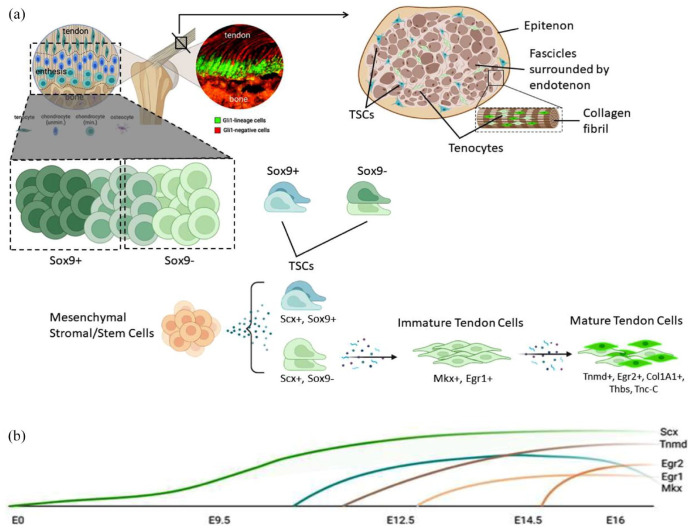
Expression of tendon markers in tenocytes during tendon development. (a) Mesenchymal cells differentiate into Scx-expressing tendon progenitor cells, which also partially express Sox9. Scx+Sox9+ progenitor cells differentiate into the tenocytes which are located near the bone in the enthesis. (b) In mouse limbs, Scx expression begins to increase at E9.5 and continues to increase until tenocyte maturation. Slight Mkx expression is detectable in the tendon at E12.5, after the emergence of Scx and robust Mkx mRNA expression at E13.5 and E14.5, stages at which the tendon progenitors undergo condensation and differentiation. Egr1 transcripts are first expressed at E12.5 in Scx domains forming tendons, and then they are expressed in long tendons at E16.5. Egr2 is first detectable in E14.5 limb tendons and is generally expressed in all limb tendons by E16.5. Tnmd is highly expressed in E14.5 and is considered a late tendon marker. Adapted from He et al.^20^

Conversely, the cartilage precursor cells of the sclerotome have an opposite role in controlling the specification of the tendon progenitors. The signal from the sclerotome activated by Sonic Hedgehog (SHH) expression, blocks Scx expression directly, through Pax1 activity, or indirectly by means of Sox5 and Sox6, which in turn, is induced by Sox9 (Figure 2). These genes are also essential in cartilage formation, with the Scx^+^/Sox9^+^ pool essential for the formation of the chondro-tendinous/ligamentous junction, which further develops into the osteo-tendinous/ligamentous junction, providing anchorage of the tendon to the muscles (Figure 3).

Although the progenitors of muscle, cartilage and tendon arise in different compartments of the somite, the differentiation of tendons depends on a combination of both activating and repressing signals from the other two compartments. Progenitors of the limb tendons develop differently from the cells that give rise to axial tendons because they are not localized within a specific subdomain in the somite (Figure 3). The initiation of Scx expression in tendon progenitor cells in the limbs does not require signals from myogenic cells (including FGFs), but they are necessary for their continued differentiation.^21^ Both FGF and TGFβ can induce tendon development.^22^ In particular, TGFβ signalling is important for the maintenance and recruitment of tendon progenitors in a paracrine or autocrine manner.^23^ Moreover, mechanical forces maintain the expression of Scx through TGFβ/Smad2/3 mediated signalling.^24^ Scx and Mkx play a pivotal role in providing the starting impulse for limb tendon formation. The expression of Mkx is detectable after the emergence of Scx, and its strongest expression is recognized in the sheath cells.^24^ The role of Mkx is essential for tendon growth regulation after tendon progenitor initiation during embryogenesis and is relevant for the regulation of the postnatal growth and maturation of collagen fibrils and other tendon-related proteins such as decorin, fibromodulin and lumican.^25^

Bone morphogenetic protein (BMP) family members, including growth and differentiation factors (GDF) isoforms GDF5, 6 and 7, also known as BMP14,13 and 12, have been implicated in tendon development^26^ and healing. The absence of these factors directly affects the biological and biomechanical cues of the neo-tendon. Of relevant importance is the mutual antagonism between BMP and FGF signalling: the activation of FGF inhibits BMP consequently promoting tendon formation, whereas the activation of BMP induces the FGF inhibition, resulting ultimately in chondrogenesis.^21^

These studies have highlighted specific growth factors and transcription factors involved in tenogenesis (Table 1) during developmental and repair processes. Although mechanical factors also seem to be essential for tendon development, understanding the link between the mechanical and biological parameters involved in tendon development, homeostasis and repair is a prerequisite for the identification of effective treatments for chronic and acute tendon injuries.

**Table 1.** Growth factors and transcription factors involved in tenogenesis.

Gene	Category	Role	Phenotype under abnormal expression	Ref.
MKX	Transcription Factor	Tendon maturation and differentiation Maintaining TSCs characteristic	• Reduced Scx expression • Decrease of ColI, Fmod, Dcn • Small collagen fibres diameter • Tendon sheath is thicker • Increase amount of cells • Reduced tensile strength • Abnormal collagen fibril structure	Liu et al.^11^, Dale et al.^26^, Otabe et al.^27^
SCX	Transcription Factor	Embryonic development of tendons induced with mechanical stimuli Early marker of tendon developmental and differentiation	• The tendon matrix results to be reduced and disorganized • Hypocellularity • Disorganized endotenon • Downregulation of Mkx • Reduced production of ColI	Liu et al.^11^, Ito et al.^28^, Gumucio et al.^29^, Mendias et al.^30^, Bagchi et al.^31^
EGR1/2	Transcription Factor	Act as molecular sensors for mechanical signals and are involved in collagen maturation	• Reduced expression of Scx, Mkx, Tnmd and ColI • Tendon mechanically weaker and fragile	Ellingson et al.^23^, Huang et al.^32^, Guerquin et al.^33^
TNMD	Glycoprotein	Later marker of tenogenesis Regulator of tenocyte proliferation and is involved in collagen fibril maturation but does not confirm an in vivo involvement of Tnmd in angiogenesis.	• Decrease tenocyte proliferation and density • Hypocellularity • Thicker collagen fibres • The deposited amounts of extracellular matrix proteins are not affected but they present a disorganized structure • Reduced self-renewal but increased senescence of mouse TSCs • Insufficient adhesion to collagen type I, and impaired ability to contract the extracellular matrix.	Lejard et al.^34^, Docheva et al.^35^, Yin et al.^36^, Liu et al.^37^, Alberton et al.^38^, Dex et al.^39^
BGN	Proteoglycan	Control Scx expression Regulate collagen fibrillogenesis and extracellular matrix assembly in tendon	• Decreased tendon stiffness • Alteration of the collagen fibrils • Increased fibres diameter	Dex et al.^40^, Cohen et al.^41^, Gordon et al.^42^, Dunkman et al.^43^
DCN	Proteoglycan	Regulate collagen fibrillogenesis and extracellular matrix assembly in tendon	• Dysfunctional regulation of fibril • Abnormal lateral association with other fibrils • Greater viscous properties • Reduction of collagen content • Reduced tendon strength • Increased stiffness	Gordon et al.^42^, Dourte et al.^44^, Roughley and Lee^45^
COL1A1	Protein	Main structural protein and ECM component	• Fibril dimension decrease	Dourte et al.^46^
COMP	Glycoprotein	Binding other ECM proteins, catalysing the polymerization of type II collagen fibrils, and regulating chondrocyte proliferation	• Chondrocyte cell death • Matrix is abnormal and easily erodes with normal physical activity	Löhler et al.^47^, Smith et al.^48^
FBN1	Proteoglycan	Formation of elastic fibres found in connective tissue	• Decrease collagen cross-link	Geng et al.^49^, Giusti and Pepe^50^
FMOD	Proteoglycan	Control Scx expression Required in early collagen fibrillogenesis to stabilize small-diameter fibril-intermediates	• Small diameter and immature collagen fibrils • Thinner collagen fibres • Non-crosslink • Reduced number of cells • Increase lumican	Dourte et al.^44^, Boregowda et al.^51^, Ezura et al.^52^
LUM	Proteoglycan	Needed at a later stage, primarily to limit the lateral growth of fibrils	• Disorganized matrix • Large diameter collagen fibres	Boregowda et al.^51^, Ezura et al.^52^, Majava et al.^53^
THBS-4	Glycoprotein	Late marker	• Disordered collagen fibrillogenesis	Zelzer et al.^54^, Bornstein et al.^55^

## Tendon structural and functional relationship

### Tendon histology

Tendons possess the mechanical strength and flexibility required to perform their pivotal role as active elements in joint stability during movement and physical exercise. The ability of tendon tissues to bear these loads originates from a hierarchical structural organization able to adapt to different mechanical stimuli. Mechanical loading can thus be viewed as a switch factor between functional tissue remodelling and the development of chronic tendon disease.^56^

The tendon is composed of two, not always physically distinct, tissue compartments. The first extrinsic compartment is a family of synovium-like fascias, that comprise the paratenon (tendon sheath), epitenon (sub-tendon sheath) and endotenon (fascicular sheath). These tissues include differentiated and progenitor cell populations related to the mesenchyme as well as the nervous, immune, and vascular system. The extrinsic compartment envelops the intrinsic one, defined as the tendon core.^56^ The tendon core is constituted of 65%–80% type I collagen. Its supramolecular assembly gives rise to fibrils, fibres, and bundles. In each stage of the organization, new features of mechanical properties are acquired. For this reason, it is important to clearly distinguish between fibrils, the basic subcellular collagen building block, and fibres, the relevant cell-scale structural units with which cells physically interact.^57^ At higher hierarchical levels, collagenous subunits are interspersed with a less fibrous, hydrated matrix, traditionally referred to as ground substance.^58^

The core tendon fibres, which feature interspersed tenocytes, are surrounded by fascicles, the fundamental functional unit within the intrinsic tendon. This structure is considered the first synovial tissue barrier (endotenon) between the intrinsic and extrinsic tendons. The capability of tendons to heal is strictly related to the interplay between the intrinsic compartment, defined by tendon cells and the multiscale arrangement of collagen assemblies, and the extrinsic compartment, which consists of synovium-like tissues connecting the immune, vascular and nervous systems.^23^

Type III collagen is the second most abundant tendon collagen, comprising up to 10% of total collagen content.^59^ It plays an important role in the development of fibrils used as a template for the rearrangement of tenocyte-synthesized fibronectin. Two types of collagens sustain type I collagen self-assembly, namely type IV collagen and type V collagen.^23,58^ The last fibrillar component is elastin, a glycoprotein involved in the recoil of the matrix after repetitive mechanical loading. Elastin is closely connected with collagen fibres: especially when fibres are subjected to load, they stretch and carry with them the elastic fibres.^60^ Once the external load has been removed, collagen fibres do not have intrinsic elasticity, so their recoil must be supported by elastin. The mechanical crosslink between the fibrillar components and the other molecules of the ECM is provided by Fibrillar Associated Collagens with Interrupted Triple Helices (FACIT).

Proteoglycans are the most abundant non-fibrous proteins in tendons, making up 1%–5% of tendon dry weight. Proteoglycans are a class of glycoproteins, consisting of a core protein attached to one or more polysaccharide chains.^59^ These side chains are termed glycosaminoglycan (GAGs) side chains and are negatively charged, so they tend to attract water contributing to the resistance against compressive mechanical forces and facilitating nutrient and metabolite diffusion. Finally, decorin as a major proteoglycan contributor to the tendon, together with biglycan, lumican and fibromodulin, belongs to the Small-Leucine rich proteoglycan class (SLRP). SRLPs have a critical time-dependent role in tendon development. In tissues such as tendons, fibromodulin may be required early in collagen fibrillogenesis to stabilize small-diameter fibril-intermediates, and lumican may be needed at a later stage, primarily to limit lateral growth of fibrils.^61^ Another aspect of no less importance is their capability to bind to growth factors, establishing a chemical interaction with the main collagen bundles.

In tendons, cell-ECM interactions maintain tissue homeostasis by generating cell signals that affect cell proliferation, differentiation, migration and adhesion. Nonetheless, the ECM plays a major role in the regulation and transmission of growth factors (TGFβ1, GDF-5/6/7, FGF).^62–68^ Hence, both the mechanical support of the ECM and the presence of specific growth factors contribute to the maintenance and differentiation of tendon stem cells, important factors to consider in tissue engineering applications.^69^

### Tendon cells

Mature tendons are normally characterized by a low cellular density. Approximately 90%–95% of the cellular content of tendons comprises tendon-specific cell types described in the literature as tenocytes and tenoblasts.^70^

Tenocytes, which are tendon-specific terminally differentiated fibroblasts, are spindle-shaped, with elongated nuclei and thin cytoplasmic protrusions anchoring the collagen fibres. They are laid between collagen fibrils and oversee the production of extracellular matrix (ECM) as well as the maintenance, repair, and remodelling of tendons. Tenogenic differentiation markers are commonly used for the identification of tenocytes. Tenoblasts are relatively round cells with large ovoid nuclei. Tenoblasts seem dominant in young tendons, and they transform into tenocytes during maturation and ageing. Interconversion between tenoblasts and tenocytes might occur, and their ratio in tendons may govern the tissue responses to various stimuli such as exercise and trauma.^70^ Tenoblasts are sometimes regarded as an activated form of tenocytes, such as in intrinsic healing of tendon injuries; the discrimination between tenocytes and tenoblasts, which is based on cell shape appearance, lacks precise molecular separation via marker gene expression.^71^ The remainder of cells in the tendon are mainly composed of chondrocytes at the pressure and insertion sites, synovial cells of the tendon sheath, capillary endothelial cells and smooth muscle cells of arterioles.^71^

Tendon cells synthesize all components of the tendon ECM with peak activity during growth and a gradual decrease during ageing.^70^ It is thought that low metabolic rates associated with anaerobic energy production which is typical of mature tendon cells can reduce the risk of ischaemia and necrosis, especially during the extended periods of tensional stresses to which tendons are usually subjected. On the other hand, this feature is a disadvantage for tendon recovery and healing.^69^

**Figure 4.**
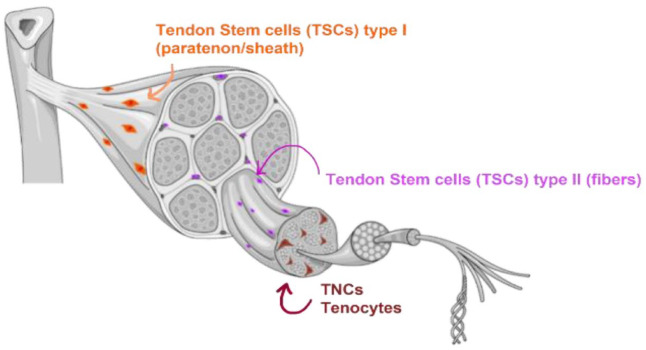
Location of different cells in tendon tissue. TSCs type I (orange) reside in the outer layer of the tendon (paratenon), TSCs type II (purple) reside in a niche in the inner part of tendons, and TNCs (brown) are aligned between fibres. Some of these subpopulations might overlap with each other, and perivascular TSCs may be present in the endotenon and peritenon (image partially created with https://www.biorender.com/).^[Bibr bibr73-20417314231196275]^

**Figure 5.**
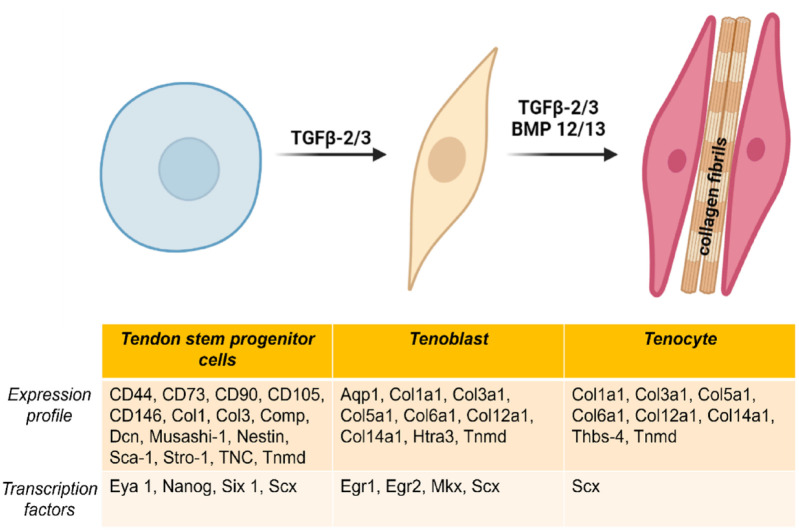
Differentiation of a TSPC to a tenocyte. The expression profile of a TSPC changes during differentiation to a tenocyte. Tenogenic differentiation is mostly driven by TGF-β2/3 and BMP12/13. Adapted from Schneider et al.^73^ (image partially created with https://www.biorender.com/).

Tendons also contain a pool of stem and progenitor cells. In 2007, Bi et al.,^72^ identified within human hamstring tendons a novel cell population of resident tendon stem/progenitor cells (TSPCs). TSPCs exhibit classical adult mesenchymal stem cell (MSC) criteria such as the presence of specific surface antigens, self-renewal, clonogenicity and three-lineage differentiation (adipogenic, osteogenic and chondrogenic),^73^ and they express tendon-related genes such as scleraxis (Scx) and tenomodulin (Tnmd) and can form tendon and enthesis-like tissues when implanted in vivo.^69^ Recently, it was proposed that there is a regional distribution of different stem/progenitor cells within the tendon, namely in the outer tendon sheet (TSPC type I) and within the tendon proper (TSPC type II)^72^(Figure 4). Comparison between these subpopulations revealed that the peritenon-derived cells have increased vascular and pericyte markers, while the tendon-proper-derived cells are more proliferative and exhibit higher levels of Scx and Tnmd.^74^ TSPCs can also be positive for some common stem cell markers, which can also be found on the surface of other mesenchymal stem cell (MSCs) types. They express Sca-1, CD44, CD90, CD90.1, CD105, CD146, Stro-1, nucleostemin, Oct-4 and SSEA-1 but not CD18, CD31, CD34, CD45, CD106, CD117, CD144 or Flk-1.^75^ Since there are no molecular markers that allow discrimination between TSPCs, tenoblasts and tenocytes (Figure 5), it is not easy to isolate pure subsets of cell populations from these differentiation stages.^73^ Ruzzini et al.^76^ isolated tendon-derived CD44+ cells, which were positive for the stem cell markers CD146 and STRO1 and reported them to be TSPCs.^77^ The exact role of TSPCs in tendon maintenance and healing is not completely understood. Hence, there is a great need for in vitro and in vivo studies demonstrating their role and precise locations.^71^

## Tendon mechanical properties

The characteristic tendon fibrillar ECM has distinct structural and biomechanical properties that facilitate the effective transmission and absorption of cyclical tensile forces while avoiding injury. These properties arise from the specialized axial and longitudinal structural organization of the collagen fibre hierarchy, which provides a scaffold for cell and macromolecule attachment. The tendon is primarily a uniaxial force-transmitting connective tissue consisting of bundles of parallel fibrillar ECM and resident tenocytes, which connect bone to muscle.^77^ Both the structure and the function of this fibrillar ECM provide biochemical and mechanical signals that cooperate in the integrated regulation of tenocyte proliferation, survival, differentiation and migration, which ultimately feedforward to maintain physiological ECM synthesis and assembly. The main purpose of collagen fibres is to resist tension, while the proteoglycans provide viscoelastic properties for the tendon. In addition to binding the fibres together, the endotenon enables fibre groups to glide over each other and carries blood vessels, nerves, and lymphatics to deeper portions of the tendon.

**Figure 6.**
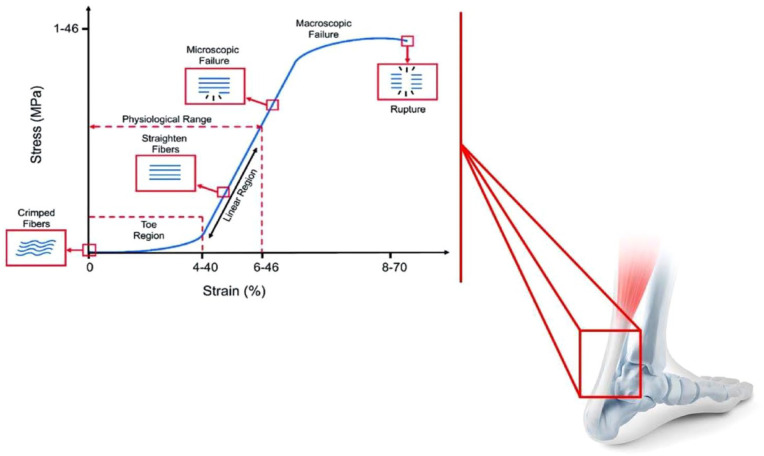
Stress–strain relationship in tendons. At low levels of stress, tendons stretch relatively easily. This is called the ‘toe’ portion of the stress–strain curve, a consequence of straightening of crimped collagen fibrils and orientation of fibres along the direction of the applied load. With higher levels of stress, the highly oriented collagen fibres respond with a linear level of strain. The slope of the linear region represents the elastic modulus of the tendon. Continuing increases in the level of stress applied to tendons and ligaments lead to irreversible changes at the interface between collagen fibres in the structure.

The initial portion of the stress-strain curve of a tendon is highly viscoelastic with a high viscous dissipation occurring during collagen fibril alignment.^10^ Depending on the type and anatomical location of the tendon, this wavy fibril pattern results in different initial mechanical properties arising from the varying angle and length of ‘crimping’.^78^ When the tendon is stretched to higher strain levels, the stiffness of the material increases rapidly as the collagen fibres are recruited, straightened, and begin to carry a major part of the load; the tendon demonstrates the transition from the toe region to the linear region in which the strain is kept below 4% and the slope of the curve is defined by Young’s modulus, which represents tendon stiffness. In the linear region of the stress-strain curve, the tendon extension and force are directly proportional to one another. The stress generated by the fibre is a function of the number of fibres available to share the load. In the third phase, all collagen fibres are straight, and the system exhibits its highest stiffness. After this stage, a decrease in the stiffness of tissue is observed, the result of a gradual mechanical failure of the collagen fibre (Figure 6).

Tissue substructures across size scales have been investigated to evidence their mechanical properties. However, the characterization of these structures outside the body, in a laboratory setting, reduces the clinical applicability of the results obtained.^79^ The fact that fibres are not stress-free constitutes an additional difficulty in the modelling of soft tissues since the strain is measured with respect to a stress-free configuration that does not exist physiologically.^80^ This stress is developed in the absence of external loads, and it is known as residual stress. Modelling residual stresses is challenging, but it is of great importance, as the material and structural properties of soft tissues can be under or over-estimated.^81,82^

The development of residual stresses is linked to cellular processes.^83^ These biological processes are a consequence of the continuous interplay between genetics and epigenetic factors. Epigenetic changes are related to the cell’s environment, and they include chemical agents and mechanical loading. Although cells contain genetic information about the structural patterns of the tissue, mechanical stimuli can condition the genetic instructions stored in the cells.^84^

Cyclic uniaxial mechanical loading of TSCs has demonstrated that proliferative capability increases and a loading magnitude-dependent differentiation effect has been observed, promoting either symmetric or asymmetric division.^85^ In the symmetric division, TSCs divide into two identical daughter cells, in the asymmetric one instead, one daughter cell is identical to the original one (self-renewal) while another becomes specialized. These observations can potentially explain how TSCs play a key role in tendinopathy development by undergoing aberrant, non-tenocyte differentiation under excessive mechanical loading conditions.^86^ When considering the uniaxial loading conditions of tendons, it has been demonstrated that tenocytes increase proliferation as well as tenogenic gene expression and protein production (Col I, Col III, TNMD, TNC-C, MMPs) in a stretching magnitude-dependent manner.^85,87,88^

Scx has a well-defined role in the development of tendons during embryogenesis as a promoter of type I collagen production. A further key protein involved in various processes, such as tenocyte proliferation, collagen organization and fibril maturation is Tnmd. Mechanical forces regulate the expression of Scx through activation of the TGF-β-/Smad2/3-mediated pathway, which, in turn, is required for the maintenance of tendon-specific ECM.^89^ In contrast, the expression of tendon-associated markers may not be influenced by applied loading protocols.^90^ In particular, there were no significant changes in Scx and Tnmd expression after loading conditions. Instead, an unexpected expression of Runt-related transcription factor 2 (Runx2), which is associated with osteogenic differentiation, has been observed. Moreover, the expression of Col1A1, Col3A1 and MMP2/3 was not upregulated in loading conditions. The resulting discrepancy can potentially be attributed to the different loading regimens used in the respective protocols. Indeed, the response of tendon cells to adapt by anabolic or catabolic processes depends on the applied frequency, magnitude, duration, and direction, all parameters applied differently by different authors.

To test whether mechanical loading of the tibia-induced molecular processes can lead to degeneration, the expression of the differentiation markers Lpl, Sox9 and Runx2 were examined as surrogates for adipogenic, chondrogenic and osteogenic differentiation, respectively.^91^ In mice, treadmill running accelerates TSC proliferation in an intensity-dependent manner. However, although the expression of Tnmd and Col1A1 was not significantly increased in the intensive and moderate treadmill running groups, the intensive running group showed up-regulation of non-tenocyte-related genes Lpl, Sox9 and Runx2. The in vivo results were confirmed also by the in vitro experiments: after 8% stretching, up-regulation of the non-tenocytes-related gene was evident. This study analysed the gene expression of MGF, an Eb form of OGF-1, a growth hormone that promotes tissue growth. A higher level of the MGF gene was expressed in tendons after moderate loading, and more after intense activity, probably related to the higher mechanical load and larger extent of ‘micro-injury’. These findings indicate that more TSCs are generated for the repair and/or remodelling of tendons in response to the demands of mechanical loads. Specifically, under mechanical loading conditions, the TSCs population in the tendon grows, providing progenitors for tenocytes and enhancing the remodelling of tendons. This may explain why appropriate moderate exercise induces anabolic effects on the tendons, including enlarged cross-sectional area, increased tendon stiffness, and enhanced tendon tensile strength. On the other hand, intense mechanical loading still enhanced MGF expression, TSCs proliferation, and increased tenocyte-related gene expression, but it can also induce degenerative changes by inducing aberrant differentiation of TSCs into non-tenocytes, which, at later stages, manifests as lipid deposition, increased number of proteoglycans, and calcified tissue in the affected tendon.^92^

Other important actors in tenocyte mechano-transduction are cytokines and integrins. Cytokines, such as interleukins (IL), tumour necrosis factor-α (TNF-α), and interferon-gamma (IFN-γ) are known to be key players in tendon disorders.^93–96^ They are released by the tendon stroma or immunoregulatory cells in response to mechanical stress or tissue injury, alter the cellular phenotype and induce changes in matrix production.^97,98^ Adequate mechanical stimuli play an essential role in tendon homeostasis, regular function, tenocyte survival and tendon healing and mechanical factors influence tendon cytokine profile.^99^ An increased amount of pro-inflammatory cytokines such as IL-1a, IL-1b, TNF-α and IFN-γ was demonstrated in inflamed native equine tendon.^100^ Over-mechano-stimulation of tendon and tenocytes leads to cytokine release such as IL-1b.^101^ Stress deprivation and absence of mechano-stimuli induced cytokine over-expression (particularly that of IL-1b, TNF-α and other cytokines such as TGFβ) and mechanical deterioration of the tissue in the patellar tendon.^102^ Ruptured tendons revealed less TNF-α expression when naturally loaded during the healing process compared with unloaded ruptured tendons in a rat Achilles tendon healing model.^103^

Integrins are cell surface receptors known to connect the ECM to the actin cytoskeleton and transmit mechanical stimuli into the cell to evoke different cell responses. The collagen-binding Itga1 and Itga2 were both downregulated in vivo following 8% mechanical loading,^104^ confirming the key role of these molecules in mechano-transduction.

## Tendon injury and healing

When a tendon is injured, its structure is disrupted, and proper function can be compromised. Unfortunately, tendon injuries are a common clinical problem, and they are broadly categorized as chronic tendinopathies or acute ruptures.^105^

On one end of the spectrum is chronic tendinopathy, initiated by biological and physical factors that include ageing, oxidative stress, and repetitive loading during intensive exercise.^106^ Indicators of chronic tendon injury range from pain, inflammation, and increased cross-sectional area to histologically observable changes including increased proteoglycan content, increased cellularity, hypervascularity, ectopic bone, cartilage nodules and disorganization of the collagen-fibril network.^107,108^ On the other end of the spectrum lie tendon ruptures.^109^ Although this type of injury may be spontaneous or induced by direct trauma and/or excessive loading, most tendon tears are preceded by histological changes consistent with chronic tendinopathy, suggesting that tendon rupture is closely associated with degeneration,^110^ though in these patients the tendinopathic changes generally remain clinically silent until the condition is manifested through an acute rupture. Investigations of tendon healing have been predominantly undertaken on transected animal tendons, and it is unclear how relevant they are to the healing of tendinopathic or ruptured human tendons.

**Figure 7.**
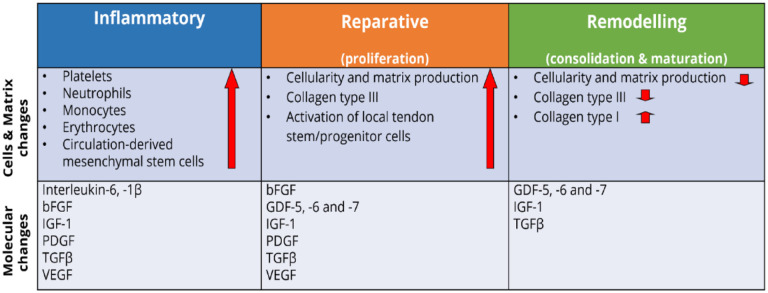
Key molecular, cellular and matrix changes occurring during the three main phases of tendon repair. Each healing stage is characterized by the involvement of different growth factors, activation of specialized cell types, and production of essential matrix proteins. Collectively, they contribute to the replacement of the initial fibrous tissue with more tendinous regenerated. Adapted from Docheva et al.^70^ and James et al.^114^

The response to a tendon injury and healing is classically considered to occur through extrinsic and intrinsic healing.^111^ In intrinsic tendon healing, the proliferation of epitenon and endotenon tenocytes takes place – Healing of the defect involves an exudative and a formative phase which, overall, are very similar to those associated with wound healing.^112^ Extrinsic healing occurs through chemotaxis of the specialized fibroblasts into the defect from the ends of the surrounding tendon sheath and, if present, synovium.^113^ The process can be divided into three overlapping phases: inflammation, repair and organization or remodelling^110,111^ (Figure 7).

In the inflammatory stage, which typically spans a few days after the injury, the wound site is infiltrated by red blood cells, white blood cells (leucocytes), and platelets secreting growth factors and endothelial chemoattractants.^110^ Cells migrate from the extrinsic peri-tendinous tissue such as the tendon sheath, periosteum, subcutaneous tissue and fascicles, and from the epitenon and endotenon.^113^ A fibrin clot provides temporary stiffness and biomechanical stability, macrophages digest necrotic debris and tenocytes are recruited to the wounded area and stimulated to proliferate, particularly in the epitenon.^115^

The second stage, the proliferative stage, begins roughly 2 days into the injury response.^110^ This phase of healing is characterized by profuse synthetic activity and is directed by macrophages and tenocytes. Macrophages, whose role shifts from phagocytic to reparative a few days after injury,^116^ release growth factors and direct cell recruitment.^110,117^ Meanwhile, tenocytes deposit a temporary, mechanically inferior matrix composed mostly of type III collagen. Tenocytes become the main cell type, and collagen is continuously synthesized over the next 5 weeks. During the fourth week, a noticeable increase in the proliferation of fibroblasts of intrinsic origin, mainly from the endotenon, takes place. These cells take over the main role in the healing process, and both synthesize and reabsorb collagen. The newly formed tissue starts to mature, and the collagen fibres increasingly align themselves along the direction of force through the tendon. This phase of repair continues for 2 months after the initial injury.^110,111^

In the third and final stage, the remodelling phase, type I collagen synthesis begins to dominate, and the ECM becomes better aligned. In addition, cell density and general synthetic activity are gradually decreased. This phase begins 1–2 months after injury and can last more than a year.^110^

Numerous bioactive molecules are involved in orchestrating the cellular response during tendon repair.^118^ A variety of growth factors are markedly upregulated following a tendon injury and are active at multiple stages of the healing process, including insulin-like growth factor-I (IGF-I), TGFβ, bFGF, platelet-derived growth factor (PDGF), vascular endothelial growth factor (VEGF), BMP and connective tissue growth factor (CTGF).^105^

Despite intensive remodelling over the following months, complete regeneration of the tendon is never achieved.^119^ The tissue replacing the defect remains hypercellular. The diameter of the collagen fibrils is altered, favouring thinner fibrils with a reduction in the biomechanical strength of the tendon. The repaired tissue appears scar-like, and never completely regains its pre-injury biomechanical properties.^110^ In tendinopathic and ruptured Achilles tendons, there is a reduction in the proportion of type I collagen, and a significant increase in the amount of type III collagen,^120^ responsible for the reduced tensile strength of the new tissue as a result of a reduced number of cross-links compared with type I collagen.^121^ Recurring micro-injuries lead to the development of hypertrophied biologically inferior tissue replacing the intact tendon.^111^

## Current status for enhancement of tendon healing and replacement

First-line therapeutic options differ for chronic and acute tendon injuries. The primary goal of tendinopathy treatment is to reduce pain, with a combination of exercises, physical therapy modalities and local and systemic pharmacological agents,^122,123^ whereas surgical techniques aim to repair ruptured tendons.^70,124^

The approach to treatment in acute soft tissue trauma relies heavily on the patient’s history, signs, and symptoms including the grade of injury, and their physical and work activities goals after therapy. Initially, clinical evaluation determines the grade of injury and the level of instability in the joint. Imaging (ultrasound and magnetic resonance imaging) assists in the diagnosis, after which the patient is considered for conservative or surgical treatment.^125^

In the case of surgical treatment, the outcomes of reconstructive surgery differ depending on the type and location of the injury. The type of suture may also play a critical role in the strength of repair and tendon autografts may be used to facilitate tendon reconstruction, particularly in cases involving tendon loss or retraction.^126^ Autografts have many advantages, such as the absence of immunological complications, rapid incorporation, and good remodelling. However, autografts also present some disadvantages, such as increased duration of operation given the time necessary for graft harvest and preparation; limited donor tendon sources; and sacrifice of the function of the donated tendon. Moreover, the neo-tendon repaired by autograft is not identical to the normal tendon and may take a long time to remodel.^127^ It should be acknowledged, however, that tendon appearance post-operatively may well exert no influence on its function.^128^

For both chronic and acute tendon injuries, exercise-based rehabilitation is indicated.^73^ Eccentric exercise therapy (involving active lengthening of muscle and tendon) has become the first-line treatment and is considered the most efficient for tendinopathy (e.g. Achilles and patellar tendon). Good-quality randomized controlled trials indicate that eccentric strengthening programmes provide 60%–90% improvement in pain and function.^127^

Mechanical loading is also integrated into clinical postoperative rehabilitation protocols. The mechanical stimulation from controlled mobilization is suggested to enhance tendon repair and remodelling by stimulating tenoblast activities (such as fibroblast proliferation and collagen synthesis and realignment), leading to increased tensile strength, increased tendon diameter and fewer adhesions compared with immobilized healing tendons.^129,130^ In contrast, immobilization following tendon injury may have a negative effect on tendon healing, as evidenced by lower tensile strength and lower strain at failure compared with control samples.^131^ Immobilization also reduces the water and proteoglycan content of tendons and increases the number of reducible collagen cross-links.^132^

The use of autologous growth factors is another therapeutic approach, which is gaining popularity in the treatment of tendon injury. Platelet-rich plasma (PRP) is a blood derivative containing high levels of growth factors, known to promote tissue healing.^133^ Because PRP is readily available and autologous, PRP therapy is considered safe and has been introduced into a clinical therapy for tendinopathy and acute tendon injury. However, the benefits of PRP injection for tendon recovery remain controversial.^134^ Extracorporeal shockwave therapy has demonstrated efficacy in randomized controlled trials^135,136^ and register studies^137^; less conventional procedures, such as phonophoresis, therapeutic ultrasonography or low-level laser therapy, are other options for the treatment of tendon injuries.^124,138^

Consequently, surgery, specific exercise-based therapy, and autologous growth factor injections are the main current treatments for tendon injuries. Unfortunately, independently of the procedure of management of tendon injury, the outcomes of both conservative treatments and surgical repair require long healing time, high re-rupture rate and scar tissue formation.^8^ In addition to being moderately effective or controversial, the underlying mechanisms of these treatments are not fully understood.^139^

## Future prospective in regenerative medicine of the tendon

Tissue engineering has now emerged as a potential alternative to tissue or organ transplantation, which involves delivering cells or therapeutics to diseased or damaged tissue to restore tissue or organ function.^14^ With this technology, tissue loss or organ failure can be treated by implantation of a tissue-engineered graft composed of some or all the three major components: cells, biomaterials/scaffold, and biomolecules (e.g. growth factors, cytokines, nucleic acids, etc.) into sites of need.^127^

A key factor in the tissue engineering approach to repair and regeneration is the availability of appropriate cells. Cell availability is crucial to access their proliferation potential, cell-to-cell signalling, bio-molecule production, and formation of extracellular matrix. Stem cells have attracted great interest in tissue engineering given their intrinsic differentiation capacity and expansion potential.^140^

Of the various stem cell types that are available for tendon and tendon-bone junction repair, mesenchymal stem cells (MSCs) are an attractive cell source as they have high proliferative potential and can differentiate into various cell types of the mesodermal lineage.^141^ The transplantation of mesenchymal stem cells (MSCs) has been reported to improve tendon, ligament and tendon-to-bone junction repair.^140–144^ MSC therapeutic area of the tendon has been focusing on specific tendon sites including the rotator cuff tendon and superficial digital flexor tendon (SDFT). Intervention through direct injection of umbilical cord-derived MSCs (UC-MSCs) has been shown to promote the healing of rotator cuff tears in rabbit.^145^ Furthermore, a single administration of adipose tissue-derived MSCs (AT-MSCs) also positively affects the collagen crosslinking and remodelling of scar tissue in SDFT lesions.^146^ Improved neovascularization during tendon healing could also be observed when horses were injected with AT-MSCs for SDFT repair.^147^

However, MSCs-like populations have now been identified from tendon tissues of various species including humans, rabbits, rats, and horses, in vitro.^72,148–150^ These tendon stem cells (TSCs) largely meet the MSC definition of the International Society for Cellular Therapy (ISCT),^151^ which is based on three criteria: adherence to plastic, specific antigen (Ag) surface expression (CD73, CD105 and CD90) and multipotent differentiation potential (through trilineage differentiation). TSCs can effectively promote the repair and regeneration of injured tendons.^89,144,152,153^ Isolated from tendon tissue, the use of tendon stem cells for tendon and tendon-bone junction repair^154^ might be advantageous since the tendon milieu is the originating environment, which may promote engraftment and differentiation of the transplanted cells.^141^

Stem cells retain self-organization capabilities.^155^ Self-organization depends on cells’ intrinsic ability to recognize, process, and react to a wide range of global and local cues, such as morphogen gradients,^156^ mechanical boundary conditions^157^ or cellular proliferation and environmental remodelling.^158^ As a result, self-organization is stimulated by developing microscale interactions that together lead to macroscale alterations. This suggests that it should also be possible to use the cell’s self-organization capacity outside of an organism, that is, in vitro, by providing an ideal and permissive environment and by including crucial spatiotemporal cues to drive multicellular responses.^159^ Together with self-organization, the ability of stem cells to self-renew and differentiate into distinct cell types from various lineages is a special quality that has been used to mimic the characteristics of organogenesis in cell culture. However, exogenous signals, like inductive growth factors, such as bone morphogenetic proteins^80,160^ (BMPs), transforming growth factor (TGF)-β and basic fibroblast growth factor^161^ (bFGF), are still required to guide cell self-organization and differentiation to obtain proper patterning. For this reason, the employment of specifically engineered cell instructive materials can be extremely beneficial. Indeed, the incorporation of adhesion ligands, such as RGD and YIGSR, within a number of hydrogel matrices (e.g. alginate, collagen, fibrin or PLA/PLGA^162^ and PCL) has provided the capacity to enhance certain cell-material responses and to increase cell survival and differentiation.^163^

Moreover, it appears that the effects of MSCs are mainly mediated by paracrine mechanisms and by the secretion of extracellular vesicles (EVs). Consequently, there is a growing interest in the clinical applications of EVs,^164^ which have the potential to be used clinically in a variety of different ways such as pharmacological delivery agents, non-invasive biomarkers for early diagnosis, and biological reagents to treat diseases as well as to enhance tissue repair and regeneration.^165^

## A biophysical approach to induce MSCs differentiation into tenocytes

Hierarchical anisotropy structures directing 3D cellular orientation play a crucial role in designing tendon tissue engineering scaffolds. As previously reported, surface nanopatterning can control the initial assembly of focal adhesions, hence guiding human mesenchymal stem cells (hMSCs) through the process of self-organization and differentiation.^155^ This process self-sustains, leading to the development of macroscopic tissues with molecular profiles and microarchitecture reminiscent of embryonic tendons.

Autologous or allogenic tendons for tendon reconstruction have routinely been applied for medical treatment, with relatively good outcomes: however, these materials encounter several problems.^152^ For this reason, a medical strategy able to reconstruct tendon ruptures involving the development of artificial tendon-like tissue with tendon-like mechanical and histological properties from human tendon cells in vitro is required. Among the multiple biochemical and biophysical cues of the tendon niche, the ECM architecture plays a key role in governing tendon cell behaviour.^166^ This underlines the importance of tendon mimetic topography on mesenchymal stem cell commitment towards a tenogenic phenotype.

While a variety of stem cells have been investigated for biomedical applications, Mesenchymal Stromal/Stem Cells are of particular interest. MSCs are multipotent stromal cells able to differentiate into several cell types. Additionally, MSCs have excellent accessibility and plasticity and promote paracrine effects. Since they can be extracted from adult tissue, mainly bone marrow and adipose tissue, they are free from ethical concerns.^10^ Bone marrow is the most widely recognized source of MSCs, but recent research has identified alternative sources of MSCs-like cells, and it has been suggested that MSCs may be present virtually in any vascularized tissue throughout the whole body.^167^

MSCs are considered immunoprivileged, given their lack of expression of several surface antigens important for T- and B-cell recognition and their capability to suppress lymphocytes.^168^ Another benefit of MSCs is that they can exert a positive influence on various blood cell types leading to an anti-inflammatory milieu during tissue repair by suppressing tissue necrosis factor (TNF)-α and interferon (INF)-γ, while stimulating the expression of suppressive cytokines like interleukin (IL)-10.^169^ hBM-MSCs displayed tenogenic differentiation under the combination of bone morphogenetic proteins and/or mechanical stimulation.^67^

However, as stated, BMSCs also have some limitations, such as painful harvesting procedures and a frequent low cell yield, reduced MSCs quality with advanced donor age, ectopic ossification, and higher risk of adhesion formation when transplanted in vivo.^170^ As the bone marrow niche contains numerous differentiated and progenitor cell types, studying the properties of MSCs in vivo is challenging. In addition to the contamination of other cell types, there is wide variation among donors, and even between different harvesting sites in the same donor. Primary MSCs cultures often display differing growth kinetics and variations in the proportion of cell populations therein. Such variability produces challenges in obtaining consistent phenotypic and functional results and may lead to incorrect interpretation of data.^171^ It should always be considered that, depending on the desired use of MSCs in distinct tissue injury treatments, the isolation protocol would need to be set up and specifically optimized to obtain MSCs with the most optimal biological properties and therapeutic potential. This should be considered in the context of the immunomodulatory properties of MSCs cultured in different microenvironments. The application of MSCs is still limited, considering, for example, the need to produce enough cells from a single donor, avoiding the senescence of the cells and the loss of therapeutic efficacy. Therefore, the promotion of cell proliferation while retaining MSC stemness in the early stages of primary cultivation is crucial. To address this issue, attempts have been made to simulate the stem cell niche in vitro.

One of these approaches has been the application of what is frequently termed reduced oxygen or hypoxia during MSC culture.^172,173^ More specifically, we can use the term physoxia to refer to the natural level of oxygen present under in vivo conditions. Physoxia could be applied to MSCs in vitro to recapitulate the influence of native local signals on the differentiation or proliferation of these.^174^ In certain types of adult stem cells (human urine stem cells, dental pulp stem cells, amniotic fluid stem cells and bone marrow stromal cells), a low oxygen concentration in vitro promotes the proliferation and maintenance of a multipotent state.^174,175^ Conversely, other investigators have demonstrated hypoxia to be a potent stimulus for differentiation into specific cell lines.^176,177^ Independently, alteration in the oxygen concentration represents a physiological stimulus, which triggers intracellular mechanisms responsible for cell death, differentiation, or cell adaptation to new environmental conditions.

Hypoxia canonically leads to the stabilization and induction of Hypoxia-inducible factor 1-α within the cells. This protein strongly influences the metabolism, proliferation, and multipotency of MSCs. It rapidly degrades upon removal of the hypoxic condition, as the degradation of the protein is oxygen dependent, with a half-life of less than 1 min. This short half-life affects the stability and expression levels during exposure to ambient air oxygen concentration and, also, during the usual cell culture routine. Moreover, hypoxic cell culture conditions may lead to a decrease in the extracellular pH (extracellular acidosis). The latter can lead to the maintenance of stemness and attenuation of the differentiation potential of MSCs.^178^

Analysis of the metabolic activities of MSCs under hypoxic conditions indicates an increase in the consumption of glucose and glutamine and the production of lactate as a consequence of switching cell metabolism from oxidative phosphorylation to anaerobic glycolysis.^179^ This shift is associated with a reduced mitochondrial transmembrane potential.^180^ A direct consequence of this metabolic activity is the increase in proliferative activity and the enhanced ability to form CFU-F. Further, an up-regulation of stemness gene expression is observed, such as Oct-4, C-myc, Nanog, Nestin and HIF-1α.^174–181^ Thus, it is possible that long-term in vitro hypoxia enhances a genetic programme that maintains the MSCs in an undifferentiated state and in parallel stimulates the expression of genes involved in the development of various cell lines.

In vivo, MSCs are integrated into specific tissue niches where their homeostasis is regulated by a balanced set of physiochemical factors,^182,183^ while in vitro only a small number of these can be simultaneously replicated. In this context, hierarchical anisotropy structures directing 3D cellular orientation play a crucial role in designing tendon tissue engineering scaffolds. As previously reported, surface nanopatterning can control the initial assembly of focal adhesions, hence guiding human mesenchymal stem cells (hMSCs) through the process of self-organization and differentiation.^155^ This process self-sustains, leading to the development of macroscopic tissues with molecular profiles and microarchitecture reminiscent of embryonic tendons.

## The role of EVs in tendon healing

Extracellular vesicles (EVs) are diverse, nanoscale membrane vesicles actively released by cells of all tissues and organs in both health and pathologies.^184^ Similar-sized vesicles can be further classified (e.g. exosomes, microvesicles) based on their biogenesis and biophysical properties. Although initially thought to be cellular debris, and thus under-appreciated, EVs are now increasingly recognized as important vehicles of intercellular communication and circulating biomarkers for disease diagnoses and prognosis.^185^

EVs are produced by all cell types and cross biological membranes/barriers to deliver payloads to target cells and organs. EVs contain surface receptors, membrane and soluble proteins, lipids, ribonucleic acids (mRNA, microRNA, tRNA, rRNA, small nucleolar RNA, small circular nucleolar RNA, piRNA, scaRNA, viral RNA, Y RNA and long non-coding RNA),^186–189^ and even genomic and mitochondrial DNAs.^189,190^ The native cargo delivery capacity of EVs has been exploited for use as drug delivery vehicles, as they are immune-compatible, noncytotoxic and non-mutagenic compared with existing viral or cellular-based therapies.^176^ EVs are highly dependent on the origin and functional status of the parent cell since they reflect their phenotypic state.^14,191^ For example, EVs derived from umbilical progenitor cells have proangiogenic effects,^192^ while platelet-derived EVs are most abundant in circulation and help to activate platelets and the formation of fibrin clots.^193^

Recent investigations revealed the potential therapeutic benefits of using MSC-derived EVs for tendinopathies and tears.^8,194^ The use of MSC-EVs as a potential therapeutic strategy for the treatment of tendon and ligament disorders has many advantages compared to cell-based therapies, including low immunogenicity, removal of the need to maintain cell viability, removal of the risk of uncontrolled proliferation and differentiation of implanted cells, and no risk of persistence from permanent grafts upon the cessation of therapy. Furthermore, MSC-EVs can be used directly, either alone or in combination with other pharmacological agents, to enhance treatment effects. The cell-targeting effects of MSC-EVs also distinguish them from other synthetic nano-vesicles and thus potentiate their role as a drug delivery nano-platform.^8^ A better understanding of the mechanisms underlying the actions of MSC-EVs on the promotion of tendon repair would facilitate the development of MSC-EVs as a new therapeutic strategy for the treatment of tendon disorders.^8^

**Figure 8.**
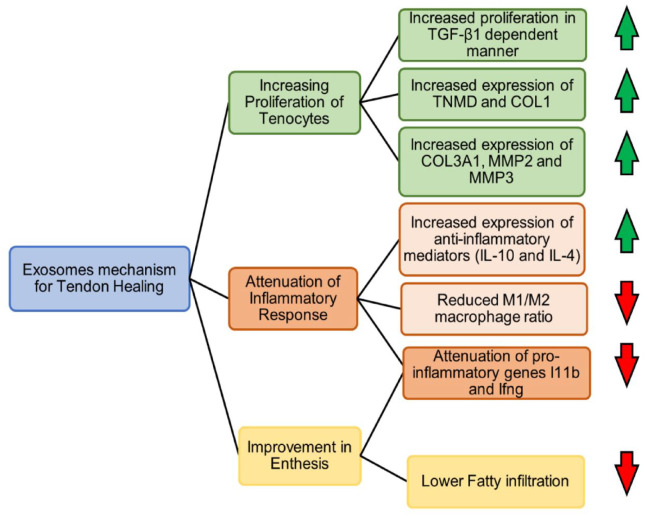
EVs mechanism of healing for tendinopathies. Schematic diagram showing the three major categories of the increasing proliferation of tenocytes, attenuation of the inflammatory response, and improvement in enthesis to explain the mechanism of healing. Adapted from Fang et al.^14^

The literature displays three prevailing methods of healing^14^: increased proliferation, migration and tenogenic differentiation of tendon stem cells (TSCs) and tenocytes,^195^ attenuating inflammatory responses,^196^ and improving the tendon–bone interface.^197^ The EVs act by the following mechanisms^14^ (Figure 8).

### Increase of the proliferation and migration of tenocytes/tendon stem cells

Tendon healing response depends on the recruitment of tenocytes and other cells from the tendon surfaces around the injured site. These tenocytes contribute to the proliferative or reparative stage of healing where they migrate, proliferate and synthesize a temporary ECM to regenerate the injured tissue.^14^

EVs derived from BM-MSCs increase cell proliferation of tenocytes in vitro, and both mRNA and protein levels of TGF-β1 are significantly higher in BMSC-derived EVs than in BMSC.^195^ Another study showed that EVs isolated from TSCs exerted wound-healing properties in vitro through the proliferation and migration of TSCs from a TGF-β pathway.^198^ Indeed, TGF-β carried by TSC-EVs, the level of which was first measured as higher than that in TSCs by immunoblotting experiments, activated the TGF-β-Smad2/3 signalling pathway and the extracellular signal-regulated kinase (ERK)1/2 signalling pathway of TSCs. The investigation of tenocyte-derived EVs reported similar results, since they contained higher protein levels of TGF-β, measured through western blotting, than their host cells and their administration facilitated the tenogenic differentiation of MSCs in a TGF-β-dependent way.^199^ The application of BMSC-derived EVs was also reported to promote the proliferation, migration and tenogenic differentiation of TSCs in vitro.^194^ The BMSCs-EVs were co-cultured with TSPCs at different concentrations for 12 h and the proliferation and migration ability of TSPCs treated with BMSCs-EVs was then detected. Consistent results through EdU+TSPCs staining, CCK8 assay, mRNA level of PCNA and wound healing assay showed that the proliferation and migration of TSPCs were significantly promoted by EVs-treated groups of cells. Moreover, BMSCs-EVs led to the induction of tendon-related genes including Tnmd, Mkx and Col1a1. Similar results were observed in vivo in the same study. The BMSC-derived EVs were embedded in fibrin hydrogels to test their repair effect on a rat patellar defect model, and 52 rats were randomly divided into 2 groups: (a) fibrin-EVs and (b) fibrin-vehicle-control of BMSCs-EVs. At histology, the fibrin-EVs group had considerably more type I collagen deposition than the fibrin-vehicle group. These results were confirmed by immunohistochemical staining of type I collagen, where the fibrin-EVs group displayed significantly higher expression of type I collagen and of Tnmd. Furthermore, the cell density, alignment, and mechanical properties in the defect region of fibrin-EVs groups were also much closer to the native tendon. BMSCs-EVs could promote the proliferation, migration and tenogenic differentiation of TSPCs.

### Attenuation of inflammatory response

Inflammation is a crucial part of wound healing and inflammatory cytokines are thought to start and maintain the injury-repair mechanism. However, ongoing inflammation results in discomfort, the creation of scar tissue, a loss of ECM organization, and deterioration of the tendon tissue.^200,201^

MSC-EVs promote tendon repair by reducing the inflammatory duration after tendon injury. EVs polarize macrophages from a pro-inflammatory M1 phenotype to a pro-regenerative M2 phenotype and attenuate the macrophage inflammatory response.^202–204^ In a mouse Achilles tendon model,^202^ EVs-educated M2-like macrophages (EEM) showed a significant improvement in tendon healing. The wound site for the EEM therapy group displayed improved biomechanical characteristics, increased tendon angiogenesis, higher numbers of endothelial cells, and improved M2/M1 macrophage ratio after 14 days. EVs also reduced the activity of nuclear factor kappa B (NFkB) at the site of tendon damage and repair, which dampens the early inflammatory response.^203^ Clinical studies showed that NF-kB is elevated in the early stages of tendinopathy and drives degeneration through mediating pro-inflammatory mediators and cytokines. In a mouse tendon-bone reconstruction model,^204^ the transplantation of BMSC-derived Ex increased the number of M2 macrophages, anti-inflammatory and chondrogenic-related factors, while it decreased the number of M1 macrophages and related proinflammatory factors. Furthermore, human BMSC-EVs reduce the expression of pro-inflammatory cytokines such as IL-1β, IL-6 and TNFα as well as stimulate the expression of anti-inflammatory IL-10^205^ and TGF-β1 in monocytic cells.^206^

### Improving the tendon-bone interface (enthesis)

The area where a tendon, ligament, or joint capsule joins to the bone is known as the enthesis. Without surgical intervention, tears or tendinopathies caused by overuse injuries, trauma, or inflammation have poor healing outcomes.^14^ However, surgery can restore function by physically reattaching tendons to the enthesis, but this process can initially be fibrous.^207^ Furthermore, the reattached tendons take longer to recover, are of lower quality, and are more susceptible to tearing or damage again.^207^

Cui et al.^197^ showed that the administration of EVs from bone marrow-derived macrophages increased the miR-21-5p gene, subsequently increasing the growth of peritendinous fibrosis and pro-fibrotic activity around the repaired tendon. These findings identified prospective targets for the prevention and therapy of tendon adhesion by showing that macrophage-derived miR-21-5p-containing EVs mediate fibrotic repair.

In a different study,^204^ BMSC-EVs were loaded on hydrogels and implanted on a mouse’s Achilles tendon injury to investigate their effects on tendon-bone healing. BMSC-EVs hydrogels increased the number of M2 macrophages and reduced the number of M1 macrophages and related proinflammatory factors (TNF-a, IL-1β and IL-6) compared to the control group. Moreover, biomechanical testing revealed that the hydrogel+BMSC-EVs group had considerably higher maximum force, strength, and elastic modulus. Similarly, adipose stem cell (ASC) EVs decreased fatty infiltration in a rabbit model of acute rotator cuff tears and improved histological and mechanical integrity, with more regenerated fibrocartilage.^208^ This study proved the anti-inflammatory actions of ASC-EVs, which reduced the infiltration of inflammatory cells, decreased the creation of fibrous scar tissue, and promoted the regeneration of the wound site.

These studies support the rationale for the development of an EV-based therapeutic approach that is highly effective for the treatment of tendon injury. Thus, the emerging role of EVs will improve the development of mechanisms, diagnoses, and therapeutic research of joint diseases. However, some challenges still exist related to the clinical applications of EVs, such as the sample size and long-term storage, the validation of the local and systemic therapeutic dose of EVs and the development of methods for selective extraction of EVs with tendon regenerative potential.

## Conclusion

Tendon injuries are a common clinical problem that results from overuse, genetic predisposition, tears, or ageing. More than 30 million tendon injuries occur annually worldwide.^209^ Tendon injury is currently managed by two approaches: (1) conservative treatment which aims to relieve pain and (2) surgical exploration, with excision and/or repair. Irrespective of the approaches used, the full restoration of tendon functions is challenging considering the high mechanical loads they are subjected to and the low cell density and vascularization that hamper the synthesis of extracellular matrix. Repeated ruptures, joint stiffness, and restricted movement are common problems encountered even after repair.^140^

The inability of the tendon to self-repair and the inefficiency of current treatment regimens have sparked the exploration of alternative treatment strategies.^140^ Biological augmentation and tissue engineering approaches are promising avenues of nonsurgical treatment presently explored.^14^ They consist mainly of applying growth factors, singly or in combination, stem cells in native or genetically modified form, and biomaterials, alone or cell-loaded, at the site of tendon damage^70^ to try to regain original tissue or organ structure and function. From the biological point of view, the implementation of a tissue-engineered tendon is based on two main steps: the finding of precursors/progenitors based on their origin and localization and the commitment and differentiation based on pivotal signalling cascades. Mesenchymal stem cells have shown great potential within regenerative medicine exhibiting differentiation capability when exposed to soluble signals like growth factors.^66,210–212^

There are mainly two problems that researchers face during the regeneration of tendinous tissues. One is to achieve the generation of a highly specialized and three-dimensional organized matrix, whose formation implies not only biological but also mechanical constraints. The second challenge is to prevent inappropriate plasticity of exogenous cells, or trans-differentiation of the local tenocytes into undesirable lineages leading to, for example, in situ adipose, cartilaginous, or bone tissue formation.^70^ To facilitate the transmission of the right signal to cells, researchers are increasingly studying the potential of EVs, which perform the role of mediators of intercellular communication by conveying biological information between cells.

Tendon tissue engineering seeks to provide alternatives to autograft by exploiting a biocompatible material that acts as a scaffold for cell reprogramming and in vivo tissue remodelling to fabricate a tendon-like tissue.^213^ Implementing EVs enriched three-dimensional scaffolds could aid in guiding and controlling cell orientation, ultimately enabling to obtaining of anisotropic tissues in vitro. Therefore, cell response results from the elaboration of multiple signals that come from the material and the de novo synthesized matrix whose structure and composition constantly change in time and space owing to cell activity. Engineering artificial platforms intended to sustain tissue production in vitro must consider the dynamic structural and compositional changes of the system. The array of signals embossed on the material surface constitutes the initial condition from which cell-cell and cell-matrix interactions establish and guide the evolution of the cells’ differentiation path.

Through examination of the results of in vivo and in vitro experiments, stem cell EVs can be considered as early diagnostic and prognostic biomarkers, as well as novel, targeted therapeutics, and drug carriers for various diseases, such as myocardial infarction, burns, and pulmonary fibrosis, with superior results, compared to using stem cells exclusively.^8,214^ Furthermore, bone marrow MSCs, TSCs and tenocytes EVs can promote macrophage polarization at the site of tendon-bone injury to eliminate inflammation, strengthen the formation of fibrocartilage to improve biomechanical properties and enhance cell proliferation, migration and tenogenic differentiation of tendon stem cells and tenocytes.^215^

## References

[1] Oliva F Gatti S Porcellini G , et al. Growth factors and tendon healing. Rotator Cuff Tear Med Sport Sci Basel Karger 2012; 57: 53–64. 10.1159/000328878 21986045

[2] Maffulli N Cuozzo F Migliorini F , et al. The tendon unit: biochemical, biomechanical, hormonal influences. J Orthop Surg 2023; 18: 311. 10.1186/s13018-023-03796-4 PMC10120196 37085854

[3] Maffulli N Khan KM Puddu G. Overuse tendon conditions: time to change a confusing terminology. Arthrosc J Arthrosc Relat Surg 1998; 14: 840–843. 10.1016/s0749-8063(98)70021-0 9848596

[4] Cook JL Purdam CR. Is tendon pathology a continuum? A pathology model to explain the clinical presentation of load-induced tendinopathy. Br J Sports Med 2009; 43: 409–416. 18812414 10.1136/bjsm.2008.051193

[5] Steinmann S Pfeifer CG Brochhausen C , et al. Spectrum of tendon pathologies: triggers, trails and end-state. Int J Mol Sci 2020; 21: 844. 32013018 10.3390/ijms21030844 PMC7037288

[6] Asahara H Inui M Lotz MK. Tendons and ligaments: connecting developmental biology to musculoskeletal disease pathogenesis. J Bone Min Res 2017; 32: 1773–1782. 10.1002/jbmr.3199 PMC5585011 28621492

[7] Bullough R Finnigan T Kay A , et al. Tendon repair through stem cell intervention: cellular and molecular approaches. Disabil Rehabil 2008; 30: 1746–1751. 18720123 10.1080/09638280701788258

[8] Lui PPY . Mesenchymal stem cell-derived extracellular vesicles for the promotion of tendon repair - an update of literature. Stem Cell Rev Rep 2021; 17: 379–389. 32785869 10.1007/s12015-020-10023-8

[9] Lamparelli EP Ciardulli MC Scala P , et al. Lipid nano-vesicles for thyroid hormone encapsulation: a comparison between different fabrication technologies, drug loading, and an in vitro delivery to human tendon stem/progenitor cells in 2D and 3D culture. Int J Pharm 2022; 624: 122007. 35820518 10.1016/j.ijpharm.2022.122007

[10] Citeroni MR Ciardulli MC Russo V , et al. In vitro innovation of tendon tissue engineering strategies. Int J Mol Sci 2020; 21: 6726. 32937830 10.3390/ijms21186726 PMC7555358

[11] Liu W Watson SS Lan Y , et al. The atypical homeodomain transcription factor Mohawk controls tendon morphogenesis. Mol Cell Biol 2010; 30: 4797–4807. 20696843 10.1128/MCB.00207-10 PMC2950547

[12] Prudêncio DA Maffulli N Migliorini F , et al. Eccentric exercise is more effective than other exercises in the treatment of mid-portion Achilles tendinopathy: systematic review and meta-analysis. BMC Sports Sci Med Rehabil 2023; 15: 9. 36698184 10.1186/s13102-023-00618-2 PMC9878810

[13] Paredes JJ Andarawis-Puri N. Therapeutics for tendon regeneration: a multidisciplinary review of tendon research for improved healing. Ann N Y Acad Sci 2016; 1383: 125–138. 27768813 10.1111/nyas.13228 PMC5560267

[14] Fang WH Agrawal DK Thankam FG. Smart exosomes”: a smart approach for tendon regeneration. Tissue Eng Part B Rev 2022; 28: 613–625. 34074136 10.1089/ten.TEB.2021.0075

[15] Heinemeier KM Schjerling P Heinemeier J , et al. Lack of tissue renewal in human adult Achilles tendon is revealed by nuclear bomb 14C. FASEB J 2013; 27: 2074–2079. 23401563 10.1096/fj.12-225599 PMC3633810

[16] Hollway GE Bryson-Richardson RJ Berger S , et al. Whole-somite rotation generates muscle progenitor cell compartments in the developing zebrafish embryo. Dev Cell 2007; 12: 207–219. 17276339 10.1016/j.devcel.2007.01.001

[17] Brent AE Schweitzer R Tabin CJ. A somitic compartment of tendon progenitors. Cell 2003; 113: 235–248. 12705871 10.1016/s0092-8674(03)00268-x

[18] Schweitzer R Chyung JH Murtaugh LC , et al. Analysis of the tendon cell fate using scleraxis, a specific marker for tendons and ligaments. Development 2001; 128: 3855–3866. 11585810 10.1242/dev.128.19.3855

[19] Delgado Caceres M Pfeifer CG Docheva D . Understanding tendons: lessons from transgenic mouse models. Stem Cells Dev 2018; 27: 1161–1174. 29978741 10.1089/scd.2018.0121 PMC6121181

[20] He P Ruan D Huang Z , et al. Comparison of tendon development versus tendon healing and regeneration. Front Cell Dev Biol 2022; 10: 821667. 35141224 10.3389/fcell.2022.821667 PMC8819183

[21] Brent AE Braun T Tabin CJ. Genetic analysis of interactions between the somitic muscle, cartilage and tendon cell lineages during mouse development. Development 2005; 132: 515–528. 15634692 10.1242/dev.01605

[22] Havis E Bonnin M-A de Lima JE , et al. TGFβ and FGF promote tendon progenitor fate and act downstream of muscle contraction to regulate tendon differentiation during chick limb development. Dev Camb Engl 2016; 143: 3839–3851. 10.1242/dev.136242 27624906

[23] Ellingson AJ Pancheri NM Schiele NR. Regulators of collagen crosslinking in developing and adult tendons. Eur Cell Mater 2022; 43: 130–152. 35380167 10.22203/eCM.v043a11 PMC9583849

[24] Liu C-F Aschbacher-Smith L Barthelery NJ , et al. What we should know before using tissue engineering techniques to repair injured tendons: a developmental biology perspective. Tissue Eng Part B Rev 2011; 17: 165–176. 21314435 10.1089/ten.teb.2010.0662 PMC3098959

[25] Liu H Zhang C Zhu S , et al. Mohawk promotes the tenogenesis of mesenchymal stem cells through activation of the TGFβ signaling pathway. Stem Cells 2015; 33: 443–455. 25332192 10.1002/stem.1866

[26] Dale TP Mazher S Webb WR , et al. Tenogenic differentiation of human embryonic stem cells. Tissue Eng Part A 2018; 24: 361–368. 28548630 10.1089/ten.TEA.2017.0017

[27] Otabe K Nakahara H Hasegawa A , et al. Transcription factor Mohawk controls tenogenic differentiation of bone marrow mesenchymal stem cells in vitro and in vivo. J Orthop Res 2015; 33(1): 1–8. 25312837 10.1002/jor.22750 PMC4294629

[28] Ito Y Toriuchi N Yoshitaka T , et al. The Mohawk homeobox gene is a critical regulator of tendon differentiation. Proc Natl Acad Sci 2010; 107: 10538–10542. 20498044 10.1073/pnas.1000525107 PMC2890854

[29] Gumucio JP Schonk MM Kharaz YA , et al. Scleraxis is required for the growth of adult tendons in response to mechanical loading. JCI Insight 2020; 5(13): e138295. 10.1172/jci.insight.138295 PMC7406294 32463804

[30] Mendias CL Bakhurin KI Faulkner JA. Tendons of myostatin-deficient mice are small, brittle, and hypocellular. Proc Natl Acad Sci USA 2008; 105: 388–393. 18162552 10.1073/pnas.0707069105 PMC2224222

[31] Bagchi RA Roche P Aroutiounova N , et al. The transcription factor scleraxis is a critical regulator of cardiac fibroblast phenotype. BMC Biol 2016; 14: 21. 26988708 10.1186/s12915-016-0243-8 PMC4794909

[32] Huang AH Lu HH Schweitzer R. Molecular regulation of tendon cell fate during development. J Orthop Res 2015; 33: 800–812. 25664867 10.1002/jor.22834

[33] Guerquin M-J Charvet B Nourissat G , et al. Transcription factor EGR1 directs tendon differentiation and promotes tendon repair. J Clin Investig 2013; 123: 3564–3576. 23863709 10.1172/JCI67521 PMC4011025

[34] Lejard V Blais F Guerquin M-J , et al. EGR1 and EGR2 involvement in vertebrate tendon differentiation. J Biol Chem 2011; 286: 5855–5867. 21173153 10.1074/jbc.M110.153106 PMC3037698

[35] Docheva D Hunziker EB Fässler R , et al. Tenomodulin is necessary for tenocyte proliferation and tendon maturation. Mol Cell Biol 2005; 25: 699–705. 15632070 10.1128/MCB.25.2.699-705.2005 PMC543433

[36] Yin H Caceres MD Yan Z , et al. Tenomodulin regulates matrix remodeling of mouse tendon stem/progenitor cells in an ex vivo collagen I gel model. Biochem Biophys Res Commun 2019; 512: 691–697. 30922565 10.1016/j.bbrc.2019.03.063

[37] Liu H Zhu S Zhang C , et al. Crucial transcription factors in tendon development and differentiation: their potential for tendon regeneration. Cell Tissue Res 2014; 356: 287–298. 24705622 10.1007/s00441-014-1834-8

[38] Alberton P Dex S Popov C , et al. Loss of tenomodulin results in reduced self-renewal and augmented senescence of tendon stem/progenitor cells. Stem Cells Dev 2015; 24: 597–609. 25351164 10.1089/scd.2014.0314 PMC4333258

[39] Dex S Lin D Shukunami C , et al. TENOgenic MODULating INsider factor: systematic assesment on the functions of tenomodulin gene. Gene 2016; 587(1): 1–17. 27129941 10.1016/j.gene.2016.04.051 PMC4897592

[40] Dex S Alberton P Willkomm L , et al. Tenomodulin is required for tendon endurance running and collagen I fibril adaptation to mechanical load. EBioMedicine 2017; 20: 240–254. 28566251 10.1016/j.ebiom.2017.05.003 PMC5478207

[41] Cohen DR Cheng CW Cheng SH , et al. Expression of two novel mouse Iroquois homeobox genes during neurogenesis. Mech Dev 2000; 91: 317–321. 10704856 10.1016/s0925-4773(99)00263-4

[42] Gordon JA Freedman BR Zuskov A , et al. Achilles tendons from decorin- and biglycan-null mouse models have inferior mechanical and structural properties predicted by an image-based empirical damage model. J Biomech 2015; 48: 2110–2115. 25888014 10.1016/j.jbiomech.2015.02.058 PMC4492865

[43] Dunkman AA Buckley MR Mienaltowski MJ , et al. The tendon injury response is influenced by Decorin and biglycan. Ann Biomed Eng 2014; 42: 619–630. 24072490 10.1007/s10439-013-0915-2 PMC3943488

[44] Dourte LM Pathmanathan L Mienaltowski MJ , et al. Mechanical, compositional, and structural properties of the mouse patellar tendon with changes in biglycan gene expression. J Orthop Res 2013; 31: 1430–1437. 23592048 10.1002/jor.22372 PMC3801205

[45] Roughley PJ Lee ER. Cartilage proteoglycans: Structure and potential functions. Microsc Res Tech 1994; 28: 385–397. 7919526 10.1002/jemt.1070280505

[46] Dourte LM Pathmanathan L Jawad AF , et al. Influence of decorin on the mechanical, compositional, and structural properties of the mouse patellar tendon. J Biomech Eng 2012; 134: 031005. 10.1115/1.4006200 PMC3705828 22482685

[47] Löhler J Timpl R Jaenisch R. Embryonic lethal mutation in mouse collagen I gene causes rupture of blood vessels and is associated with erythropoietic and mesenchymal cell death. Cell 1984; 38: 597–607. 6467375 10.1016/0092-8674(84)90514-2

[48] Smith RK Zunino L Webbon PM , et al. The distribution of cartilage oligomeric matrix protein (COMP) in tendon and its variation with tendon site, age and load. Matrix Biol 1997; 16: 255–271. 9501326 10.1016/s0945-053x(97)90014-7

[49] Geng H Carlsen S Nandakumar KS , et al. Cartilage oligomeric matrix protein deficiency promotes early onset and the chronic development of collagen-induced arthritis. Arthritis Res Ther 2008; 10: R134. 10.1186/ar2551 PMC2656236 19014566

[50] Giusti B Pepe G. Fibrillins in tendon. Front Aging Neurosci 2016; 8: 237. 27812333 10.3389/fnagi.2016.00237 PMC5071311

[51] Boregowda R Paul E White J , et al. Bone and soft connective tissue alterations result from loss of fibrillin-2 expression. Matrix Biol 2008; 27: 661–666. 18838118 10.1016/j.matbio.2008.09.579

[52] Ezura Y Chakravarti S Oldberg A , et al. Differential expression of Lumican and fibromodulin regulate collagen fibrillogenesis in developing mouse tendons. J Cell Biol 2000; 151: 779–788. 11076963 10.1083/jcb.151.4.779 PMC2169450

[53] Majava M Bishop PN Hägg P , et al. Novel mutations in the small leucine-rich repeat protein/proteoglycan (SLRP) genes in high myopia. Hum Mutat 2007; 28: 336–344. 17117407 10.1002/humu.20444

[54] Zelzer E Blitz E Killian ML , et al. Tendon-to-bone attachment: from development to maturity. Birth Defects Res C Embryo Today 2014; 102: 101–112. 24677726 10.1002/bdrc.21056 PMC4076491

[55] Bornstein P Kyriakides TR Yang Z , et al. Thrombospondin 2 modulates collagen fibrillogenesis and angiogenesis. J Investig Dermatol Symp Proc 2000; 5: 61–66. 10.1046/j.1087-0024.2000.00005.x 11147677

[56] Kyriakides TR Zhu YH Smith LT , et al. Mice that lack thrombospondin 2 display connective tissue abnormalities that are associated with disordered collagen fibrillogenesis, an increased vascular density, and a bleeding diathesis. J Cell Biol 1998; 140: 419–430. 9442117 10.1083/jcb.140.2.419 PMC2132586

[57] Snedeker JG Foolen J. Tendon injury and repair - A perspective on the basic mechanisms of tendon disease and future clinical therapy. Acta Biomater 2017; 63: 18–36. 28867648 10.1016/j.actbio.2017.08.032

[58] Zafar MS Mahmood A Maffulli N. Basic Science and clinical aspects of Achilles tendinopathy. Sports Med Arthrosc 2009; 17: 190–197. 19680116 10.1097/JSA.0b013e3181b37eb7

[59] Thorpe CT Karunaseelan KJ Ng Chieng Hin J , et al. Distribution of proteins within different compartments of tendon varies according to tendon type. J Anat 2016; 229: 450–458. 27113131 10.1111/joa.12485 PMC4974547

[60] Thorpe CT Screen HRC . Tendon structure and composition. In: Ackermann PW Hart DA (eds) Metabolic influences on risk for tendon disorders. Cham: Springer International Publishing, 2016, pp.3–10.

[61] Artells R Pruna R Dellal A , et al. Elastin: a possible genetic biomarker for more severe ligament injuries in elite soccer. A pilot study. Muscles Ligaments Tendons J 2016; 6: 188–192. 27900291 10.11138/mltj/2016.6.2.188 PMC5115249

[62] Chakravarti S. Functions of lumican and fibromodulin: lessons from knockout mice. Glycoconj J 2002; 19: 287–293. 12975607 10.1023/A:1025348417078

[63] Testa S Costantini M Fornetti E , et al. Combination of biochemical and mechanical cues for tendon tissue engineering. J Cell Mol Med 2017; 21: 2711–2719. 28470843 10.1111/jcmm.13186 PMC5661263

[64] Zhang B Luo Q Deng B , et al. Construction of tendon replacement tissue based on collagen sponge and mesenchymal stem cells by coupled mechano-chemical induction and evaluation of its tendon repair abilities. Acta Biomater 2018; 74: 247–259. 29702290 10.1016/j.actbio.2018.04.047

[65] Raabe O Shell K Fietz D , et al. Tenogenic differentiation of equine adipose-tissue-derived stem cells under the influence of tensile strain, growth differentiation factors and various oxygen tensions. Cell Tissue Res 2013; 352: 509–521. 23430474 10.1007/s00441-013-1574-1

[66] Govoni M Berardi AC Muscari C , et al. An engineered multiphase three-dimensional microenvironment to ensure the controlled delivery of cyclic strain and human growth differentiation factor 5 for the tenogenic commitment of human bone marrow mesenchymal stem cells. Tissue Eng Part A 2017; 23: 811–822. 28401805 10.1089/ten.TEA.2016.0407

[67] Tan S-L Ahmad RE Ahmad TS , et al. Effect of growth differentiation factor 5 on the proliferation and tenogenic differentiation potential of human mesenchymal stem cells in vitro. Cells Tissues Organs 2012; 196: 325–338. 22653337 10.1159/000335693

[68] Ciardulli MC Marino L Lamparelli EP , et al. Dose-response tendon-specific markers induction by growth differentiation factor-5 in human bone marrow and umbilical cord mesenchymal stem cells. Int J Mol Sci 2020; 21: 5905. 32824547 10.3390/ijms21165905 PMC7460605

[69] Citeroni MR Mauro A Ciardulli MC , et al. Amnion-derived teno-inductive secretomes: a novel approach to foster tendon differentiation and regeneration in an Ovine model. Front Bioeng Biotechnol 2021; 9: 649288. 33777919 10.3389/fbioe.2021.649288 PMC7991318

[70] Docheva D Müller SA Majewski M , et al. Biologics for tendon repair. Adv Drug Deliv Rev 2015; 84: 222–239. 25446135 10.1016/j.addr.2014.11.015 PMC4519231

[71] Chuen FS Chuk CY Ping WY , et al. Immunohistochemical characterization of cells in adult human patellar tendons. J Histochem Cytochem 2004; 52: 1151–1157. 15314082 10.1369/jhc.3A6232.2004

[72] Bi Y Ehirchiou D Kilts TM , et al. Identification of tendon stem/progenitor cells and the role of the extracellular matrix in their niche. Nat Med 2007; 13: 1219–1227. 17828274 10.1038/nm1630

[bibr73-20417314231196275] Schneider M Angele P Järvinen TAH , et al. Rescue plan for Achilles: therapeutics steering the fate and functions of stem cells in tendon wound healing. Adv Drug Deliv Rev 2018; 129: 352–375. 29278683 10.1016/j.addr.2017.12.016

[74] Mienaltowski MJ Adams SM Birk DE. Regional differences in stem cell/progenitor cell populations from the mouse Achilles tendon. Tissue Eng Part A 2013; 19: 199–210. 22871316 10.1089/ten.tea.2012.0182 PMC3530943

[75] Kannus P. Structure of the tendon connective tissue. Scand J Med Sci Sports 2000; 10: 312–320. 11085557 10.1034/j.1600-0838.2000.010006312.x

[76] Ruzzini L Abbruzzese F Rainer A , et al. Characterization of age-related changes of tendon stem cells from adult human tendons. Knee Surg Sports Traumatol Arthrosc 2014; 22: 2856–2866. 23503946 10.1007/s00167-013-2457-4

[77] Maganaris CN Narici MV Maffulli N. Biomechanics of the Achilles tendon. Disabil Rehabil 2008; 30: 1542–1547. 18720120 10.1080/09638280701785494

[78] Li Y Wu T Liu S. Identification and distinction of tenocytes and tendon-derived stem cells. Front Cell Dev Biol 2021; 9: 629515. 33937230 10.3389/fcell.2021.629515 PMC8085586

[79] Wang JH-C Guo Q Li B . Tendon biomechanics and mechanobiology—a minireview of basic concepts and recent advancements. J Hand Ther 2012; 25: 133–141. 21925835 10.1016/j.jht.2011.07.004 PMC3244520

[80] Chen D Zhao M Mundy GR. Bone morphogenetic proteins. Growth Factors 2004; 22: 233–241. 15621726 10.1080/08977190412331279890

[81] Ambrosi D Preziosi L Vitale G. The insight of mixtures theory for growth and remodeling. Z Für Angew Math Phys 2010; 61: 177–191.

[82] Rausch MK Kuhl E. On the effect of prestrain and residual stress in thin biological membranes. J Mech Phys Solids 2013; 61: 1955–1969. 23976792 10.1016/j.jmps.2013.04.005 PMC3747014

[83] Pajic-Lijakovic I Milivojevic M. Collective cell migration and residual stress accumulation: Rheological consideration. J Biomech 2020; 108: 109898. 32636009 10.1016/j.jbiomech.2020.109898

[84] Tang Y Wang Z Xiang L , et al. Functional biomaterials for tendon/ligament repair and regeneration. Regen Biomater 2022; 9: rbac062. 10.1093/rb/rbac062 PMC9514853 36176715

[85] Ventre M Causa F Netti PA. Determinants of cell-material crosstalk at the interface: towards engineering of cell instructive materials. J R Soc Interface 2012; 9: 2017–2032. 22753785 10.1098/rsif.2012.0308 PMC3405766

[86] Zhang J Wang JH. Mechanobiological response of tendon stem cells: implications of tendon homeostasis and pathogenesis of tendinopathy. J Orthop Res 2010; 28: 639–643. 19918904 10.1002/jor.21046

[87] Gomes M Reis RL Rodrigues M. Tendon regeneration: understanding tissue physiology and development to engineer functional substitutes. Academic Press, 2015.

[88] Subramanian G Stasuk A Elsaadany M , et al. Effect of uniaxial tensile cyclic loading regimes on matrix organization and tenogenic differentiation of adipose-derived stem cells encapsulated within 3D collagen scaffolds. Stem Cells Int 2017; 2017: 6072406. 29375625 10.1155/2017/6072406 PMC5742457

[89] Zhang J Li B Wang JH. The role of engineered tendon matrix in the stemness of tendon stem cells in vitro and the promotion of tendon-like tissue formation in vivo. Biomaterials 2011; 32: 6972–6981. 21703682 10.1016/j.biomaterials.2011.05.088 PMC3148341

[90] Maeda T Sakabe T Sunaga A , et al. Conversion of mechanical force into TGF-β-Mediated biochemical signals. Curr Biol 2011; 21: 933–941. 21600772 10.1016/j.cub.2011.04.007 PMC3118584

[91] Venkatesan JK Frisch J Rey-Rico A , et al. Impact of mechanical stimulation on the chondrogenic processes in human bone marrow aspirates modified to overexpress sox9 via rAAV vectors. J Exp Orthop 2017; 4: 22. 28634835 10.1186/s40634-017-0097-1 PMC5478551

[92] Fleischhacker V Klatte-Schulz F Minkwitz S , et al. In vivo and in vitro mechanical loading of mouse Achilles tendons and Tenocytes-A pilot study. Int J Mol Sci 2020; 21: 1313. 32075290 10.3390/ijms21041313 PMC7072865

[93] Zhang J Wang JH-C . The effects of mechanical loading on tendons - an in vivo and in vitro model study. PLoS One 2013; 8: e71740. 10.1371/journal.pone.0071740 PMC3747237 23977130

[94] Sziksz E Pap D Lippai R , et al. Fibrosis related inflammatory mediators: role of the IL-10 cytokine family. Mediators Inflamm 2015; 2015: 764641. 26199463 10.1155/2015/764641 PMC4495231

[95] Schulze-Tanzil G Al-Sadi O Wiegand E , et al. The role of pro-inflammatory and immunoregulatory cytokines in tendon healing and rupture: new insights. Scand J Med Sci Sports 2011; 21: 337–351. 21210861 10.1111/j.1600-0838.2010.01265.x

[96] Medzhitov R. Origin and physiological roles of inflammation. Nature 2008; 454: 428–435. 18650913 10.1038/nature07201

[97] Ciardulli MC Scala P Giudice V , et al. Stem cells from healthy and tendinopathic human tendons: morphology, collagen and cytokines expression and their response to T3 thyroid hormone. Cells 2022; 11: 2545. 36010622 10.3390/cells11162545 PMC9406581

[98] Morita W Dakin SG Snelling SJB , et al. Cytokines in tendon disease. Bone Jt Res 2017; 6: 656–664. 10.1302/2046-3758.612.BJR-2017-0112.R1 PMC5935810 29203638

[99] Ciardulli MC Marino L Lovecchio J , et al. Tendon and cytokine marker expression by human bone marrow mesenchymal stem cells in a hyaluronate/poly-lactic-Co-glycolic acid (PLGA)/fibrin three-dimensional (3D) scaffold. Cells 2020; 9: 1268. 32443833 10.3390/cells9051268 PMC7291129

[100] Arnoczky SP Lavagnino M Egerbacher M. The mechanobiological aetiopathogenesis of tendinopathy: is it the over-stimulation or the under-stimulation of tendon cells? Int J Exp Pathol 2007; 88: 217–226. 17696902 10.1111/j.1365-2613.2007.00548.x PMC2517314

[101] Hosaka Y Kirisawa R Yamamoto E , et al. Localization of cytokines in tendinocytes of the superficial digital flexor tendon in the horse. J Vet Med Sci 2002; 64: 945–947. 12419874 10.1292/jvms.64.945

[102] Tsuzaki M Bynum D Almekinders L , et al. ATP modulates load-inducible IL-1β, COX 2, and MMP-3 gene expression in human tendon cells. J Cell Biochem 2003; 89: 556–562. 12761889 10.1002/jcb.10534

[103] Uchida H Tohyama H Nagashima K , et al. Stress deprivation simultaneously induces over-expression of interleukin-1beta, tumor necrosis factor-alpha, and transforming growth factor-beta in fibroblasts and mechanical deterioration of the tissue in the patellar tendon. J Biomech 2005; 38: 791–798. 15713300 10.1016/j.jbiomech.2004.05.009

[104] Eliasson P Andersson T Aspenberg P . Rat Achilles tendon healing: mechanical loading and gene expression. J Appl Physiol Bethesda Md 1985 2009; 107: 399–407. 10.1152/japplphysiol.91563.2008 19541731

[105] Popov C Burggraf M Kreja L , et al. Mechanical stimulation of human tendon stem/progenitor cells results in upregulation of matrix proteins, integrins and MMPs, and activation of p38 and ERK1/2 kinases. BMC Mol Biol 2015; 16: 6. 25880261 10.1186/s12867-015-0036-6 PMC4373449

[106] Aicale R Tarantino D Maffulli N. Overuse injuries in sport: a comprehensive overview. J Orthop Surg 2018; 13: 309. 10.1186/s13018-018-1017-5 PMC6282309 30518382

[107] Yang G Rothrauff BB Tuan RS. Tendon and ligament regeneration and repair: clinical relevance and developmental paradigm. Birth Defects Res C Embryo Today 2013; 99: 203–222. 24078497 10.1002/bdrc.21041 PMC4041869

[108] Sharma P Maffulli N. Tendon injury and tendinopathy: healing and repair. J Bone Joint Surg Am 2005; 87: 187–202. 15634833 10.2106/JBJS.D.01850

[109] Sharma P Maffulli N. Biology of tendon injury: healing, modeling and remodeling. J Musculoskelet Neuronal Interact 2006; 6: 181–190. 16849830

[110] Kannus P Józsa L. Histopathological changes preceding spontaneous rupture of a tendon. A controlled study of 891 patients. J Bone Joint Surg Am 1991; 73: 1507–1525. 1748700

[111] Voleti PB Buckley MR Soslowsky LJ. Tendon healing: repair and regeneration. Annu Rev Biomed Eng 2012; 14: 47–71. 22809137 10.1146/annurev-bioeng-071811-150122

[112] Maffulli N Moller HD Evans CH. Tendon healing: can it be optimised? Br J Sports Med 2002; 36: 315–316. 12351325 10.1136/bjsm.36.5.315 PMC1724539

[113] Gigante A Specchia N Rapali S , et al. Fibrillogenesis in tendon healing: an experimental study. Boll Della Soc Ital Biol Sper 1996; 72: 203–210. 9009059

[114] James R Kesturu G Balian G , et al. Tendon: biology, biomechanics, repair, growth factors, and evolving treatment options. J Hand Surg 2008; 33: 102–112. 10.1016/j.jhsa.2007.09.007 18261674

[115] Wang ED. Tendon repair. J Hand Ther 1998; 11: 105–110. 9602966 10.1016/s0894-1130(98)80006-9

[116] Chisari E Rehak L Khan WS , et al. The role of the immune system in tendon healing: a systematic review. Br Med Bull 2020; 133: 49–64. 32163543 10.1093/bmb/ldz040

[117] Gelberman RH Steinberg D Amiel D , et al. Fibroblast chemotaxis after tendon repair. J Hand Surg 1991; 16: 686–693. 10.1016/0363-5023(91)90195-h 1880367

[118] Massimino ML Rapizzi E Cantini M , et al. ED2+ macrophages increase selectively myoblast proliferation in muscle cultures. Biochem Biophys Res Commun 1997; 235: 754–759. 9207234 10.1006/bbrc.1997.6823

[119] Chisari E Rehak L Khan WS , et al. Tendon healing is adversely affected by low-grade inflammation. J Orthop Surg 2021; 16: 700. 10.1186/s13018-021-02811-w PMC8642928 34863223

[120] Andia I Sanchez M Maffulli N. Tendon healing and platelet-rich plasma therapies. Expert Opin Biol Ther 2010; 10: 1415–1426. 20718690 10.1517/14712598.2010.514603

[121] Maffulli N Ewen SWB Waterston SW , et al. Tenocytes from ruptured and tendinopathic achilles tendons produce greater quantities of type III collagen than tenocytes from normal achilles tendons. An in vitro model of human tendon healing. Am J Sports Med 2000; 28: 499–505. 10921640 10.1177/03635465000280040901

[122] Maffulli N Longo UG Denaro V. Novel approaches for the management of tendinopathy. J Bone Joint Surg Am 2010; 92: 2604–2613. 21048180 10.2106/JBJS.I.01744

[123] Aicale R Bisaccia RD Oliviero A , et al. Current pharmacological approaches to the treatment of tendinopathy. Expert Opin Pharmacother 2020; 21: 1467–1477. 32511031 10.1080/14656566.2020.1763306

[124] Childress MA Beutler A. Management of chronic tendon injuries. Am Fam Physician 2013; 87: 486–490. 23547590

[125] Thaker H Sharma AK. Engaging stem cells for customized tendon regeneration. Stem Cells Int 2012; 2012: 309187. 22685473 10.1155/2012/309187 PMC3363009

[126] Schachter AK White BJ Namkoong S , et al. Revision reconstruction of a Pectoralis major tendon rupture using hamstring autograft: A Case Report. Am J Sports Med 2006; 34: 295–298. 16170043 10.1177/0363546505278697

[127] Yin Z Chen X Chen JL , et al. Stem cells for tendon tissue engineering and regeneration. Expert Opin Biol Ther 2010; 10: 689–700. 20367125 10.1517/14712591003769824

[128] Maffulli N Nilsson Helander K Migliorini F. Tendon appearance at imaging may be altered, but it may not indicate pathology. Knee Surg Sports Traumatol Arthrosc 2023; 31: 1625–1628. 36800008 10.1007/s00167-023-07339-6

[129] Kannus P. Tendons–a source of major concern in competitive and recreational athletes. Scand J Med Sci Sports 1997; 7: 53–54. 9211603

[130] Wang JH. Mechanobiology of tendon. J Biomech 2006; 39: 1563–1582. 16000201 10.1016/j.jbiomech.2005.05.011

[131] Yamamoto E Hayashi K Yamamoto N. Mechanical properties of collagen fascicles from stress-shielded patellar tendons in the rabbit. Clin Biomech 1999; 14: 418–425. 10.1016/s0268-0033(99)00006-6 10521624

[132] Akeson WH Amiel D Mechanic GL , et al. Collagen cross-linking alterations in joint contractures: changes in the reducible cross-links in periarticular connective tissue collagen after nine weeks of immobilization. Connect Tissue Res 1977; 5: 15–19. 141358 10.3109/03008207709152607

[133] Wang JH. Can PRP effectively treat injured tendons? Muscles Ligaments Tendons J 2014; 4: 35–37. 24932445 PMC4049648

[134] Jeong DU Lee C-R Lee JH , et al. Clinical applications of platelet-rich plasma in patellar tendinopathy. Biomed Res Int 2014; 2014: 249498. 25136568 10.1155/2014/249498 PMC4127290

[135] Rompe JD Furia J Maffulli N. Eccentric loading versus eccentric loading plus shock-wave treatment for midportion Achilles tendinopathy: a randomized controlled trial. Am J Sports Med 2009; 37: 463–470. 19088057 10.1177/0363546508326983

[136] Schmitz C Császár NB Milz S , et al. Efficacy and safety of extracorporeal shock wave therapy for orthopedic conditions: a systematic review on studies listed in the PEDro database. Br Med Bull 2015; 116: 115–138. 26585999 10.1093/bmb/ldv047 PMC4674007

[137] Maffulli G Hemmings S Maffulli N. Assessment of the effectiveness of extracorporeal shock wave therapy (ESWT) for soft tissue injuries (ASSERT): an online database protocol. Transl Med UniSa 2014; 10: 46–51. 25147767 PMC4140430

[138] Kaux J-F Forthomme B Goff CL , et al. Current opinions on tendinopathy. Am J Sports Sci Med 2011; 10: 238–253. PMC3761855 24149868

[139] Nourissat G Berenbaum F Duprez D. Tendon injury: from biology to tendon repair. Nat Rev Rheumatol 2015; 11: 223–233. 25734975 10.1038/nrrheum.2015.26

[140] Lui PP. Stem cell technology for tendon regeneration: current status, challenges, and future research directions. Stem Cells Cloning Adv Appl 2015; 8: 163–174. 10.2147/SCCAA.S60832 PMC4685888 26715856

[141] Lui PP Wong OT. Tendon stem cells: experimental and clinical perspectives in tendon and tendon-bone junction repair. Muscles Ligaments Tendons J 2012; 2: 163–168. 23738293 PMC3666522

[142] Hevesi M LaPrade M Saris DBF , et al. Stem cell treatment for ligament repair and reconstruction. Curr Rev Musculoskelet Med 2019; 12: 446–450. 31625113 10.1007/s12178-019-09580-4 PMC6942090

[143] Lacitignola L Crovace A Rossi G , et al. Cell therapy for tendinitis, experimental and clinical report. Vet Res Commun 2008; 32(Suppl 1): S33–S38. 10.1007/s11259-008-9085-3 18686004

[144] Lui PP Chan KM. Tendon-derived stem cells (TDSCs): from basic science to potential roles in tendon pathology and tissue engineering applications. Stem Cell Rev Rep 2011; 7: 883–897. 21611803 10.1007/s12015-011-9276-0

[145] Park G-Y Kwon DR Lee SC. Regeneration of full-thickness rotator cuff tendon tear after ultrasound-guided injection with umbilical cord blood-derived mesenchymal stem cells in a rabbit model. Stem Cells Transl Med 2015; 4: 1344–1351. 26371340 10.5966/sctm.2015-0040 PMC4622405

[146] Geburek F Roggel F van Schie HTM , et al. Effect of single intralesional treatment of surgically induced equine superficial digital flexor tendon core lesions with adipose-derived mesenchymal stromal cells: a controlled experimental trial. Stem Cell Res Ther 2017; 8: 129. 28583184 10.1186/s13287-017-0564-8 PMC5460527

[147] Conze P van Schie HT van Weeren R , et al. Effect of autologous adipose tissue-derived mesenchymal stem cells on neovascularization of artificial equine tendon lesions. Regen Med 2014; 9: 743–757. 25431911 10.2217/rme.14.55

[148] Rui Y-F Lui PP Li G , et al. Isolation and characterization of multipotent rat tendon-derived stem cells. Tissue Eng Part A 2010; 16: 1549–1558. 20001227 10.1089/ten.TEA.2009.0529

[149] Lovati AB Corradetti B Lange Consiglio A , et al. Characterization and differentiation of equine tendon-derived progenitor cells. J Biol Regul Homeost Agents 2011; 25: S75–S84. 22051173

[150] Zhang J Wang JH. Characterization of differential properties of rabbit tendon stem cells and tenocytes. BMC Musculoskelet Disord 2010; 11: 10. 20082706 10.1186/1471-2474-11-10 PMC2822826

[151] Dominici M Le Blanc K Mueller I , et al. Minimal criteria for defining multipotent mesenchymal stromal cells. The International Society for Cellular Therapy position statement. Cytotherapy 2006; 8: 315–317. 16923606 10.1080/14653240600855905

[152] Yang Z Cao H Gao S , et al. Effect of tendon stem cells in chitosan/β-glycerophosphate/collagen hydrogel on Achilles tendon healing in a rat model. Med Sci Monit 2017; 23: 4633–4643. 28951538 10.12659/MSM.906747 PMC6266537

[153] Tan C Lui PPY Lee YW , et al. Scx-Transduced tendon-derived stem cells (TDSCs) promoted better tendon repair compared to mock-transduced cells in a rat patellar tendon window injury model. PLoS One 2014; 9: e97453. 10.1371/journal.pone.0097453 PMC4022525 24831949

[154] Baldino L Cardea S Maffulli N , et al. Regeneration techniques for bone-to-tendon and muscle-to-tendon interfaces reconstruction. Br Med Bull 2016; 117: 25–37. 26837850 10.1093/bmb/ldv056

[155] Iannone M Ventre M Formisano L , et al. Nanoengineered surfaces for focal adhesion guidance trigger mesenchymal stem cell self-organization and Tenogenesis. Nano Lett 2015; 15: 1517–1525. 25699511 10.1021/nl503737k

[156] Sagner A Briscoe J. Morphogen interpretation: concentration, time, competence, and signaling dynamics. WIREs Dev Biol 2017; 6: e271. 10.1002/wdev.271 PMC5516147 28319331

[157] Anlaş AA Nelson CM. Tissue mechanics regulates form, function, and dysfunction. Curr Opin Cell Biol 2018; 54: 98–105. 29890398 10.1016/j.ceb.2018.05.012 PMC6214752

[158] Haupt A Minc N. How cells sense their own shape - mechanisms to probe cell geometry and their implications in cellular organization and function. J Cell Sci 2018; 131. jcs214015. 10.1242/jcs.214015 29581183

[159] Brassard JA Lutolf MP. Engineering stem cell self-organization to build better organoids. Cell Stem Cell 2019; 24: 860–876. 31173716 10.1016/j.stem.2019.05.005

[160] Kay AG Dale TP Akram KM , et al. BMP2 repression and optimized culture conditions promote human bone marrow-derived mesenchymal stem cell isolation. Regen Med 2015; 10: 109–125. 25835477 10.2217/rme.14.67

[161] Kraus TM Imhoff FB Reinert J , et al. Stem cells and bFGF in tendon healing: Effects of lentiviral gene transfer and long-term follow-up in a rat Achilles tendon defect model. BMC Musculoskelet Disord 2016; 17: 148. 27048602 10.1186/s12891-016-0999-6 PMC4822291

[162] Palazzo I Lamparelli EP Ciardulli MC , et al. Supercritical emulsion extraction fabricated PLA/PLGA micro/nano carriers for growth factor delivery: release profiles and cytotoxicity. Int J Pharm 2021; 592: 120108. 33238193 10.1016/j.ijpharm.2020.120108

[163] Leach JK. Multifunctional cell-instructive materials for tissue regeneration. Regen Med 2006; 1: 447–455. 17465837 10.2217/17460751.1.4.447

[164] Gupta A Cady C Fauser A-M , et al. Cell-free stem cell-derived extract formulation for regenerative medicine applications. Int J Mol Sci 2020; 21: 9364. 33316880 10.3390/ijms21249364 PMC7763336

[165] Youssef El Baradie KB Hamrick MW. Therapeutic application of extracellular vesicles for musculoskeletal repair & regeneration. Connect Tissue Res 2021; 62: 99–114. 32602385 10.1080/03008207.2020.1781102

[166] Dede Eren A Vermeulen S Schmitz TC , et al. The loop of phenotype: Dynamic reciprocity links tenocyte morphology to tendon tissue homeostasis. Acta Biomater 2023; 163: 275–286. 35584748 10.1016/j.actbio.2022.05.019

[167] Crisan M Yap S Casteilla L , et al. A perivascular origin for mesenchymal stem cells in multiple human organs. Cell Stem Cell 2008; 3: 301–313. 18786417 10.1016/j.stem.2008.07.003

[168] Niemeyer P Szalay K Luginbühl R , et al. Transplantation of human mesenchymal stem cells in a non-autogenous setting for bone regeneration in a rabbit critical-size defect model. Acta Biomater 2010; 6: 900–908. 19766744 10.1016/j.actbio.2009.09.007

[169] Aggarwal S Pittenger MF. Human mesenchymal stem cells modulate allogeneic immune cell responses. Blood 2005; 105: 1815–1822. 15494428 10.1182/blood-2004-04-1559

[170] Harris MT Butler DL Boivin GP , et al. Mesenchymal stem cells used for rabbit tendon repair can form ectopic bone and express alkaline phosphatase activity in constructs. J Orthop Res 2004; 22: 998–1003. 15304271 10.1016/j.orthres.2004.02.012

[171] Prideaux M Wright CS Noonan ML , et al. Generation of two multipotent mesenchymal progenitor cell lines capable of osteogenic, mature osteocyte, adipogenic, and chondrogenic differentiation. Sci Rep 2021; 11: 22593. 34799645 10.1038/s41598-021-02060-1 PMC8605002

[172] Forsyth NR Steeg R Ahmad M , et al. Mimicking physiological oxygen in cell cultures. In: Kasper C Charwat V Lavrentieva A (eds) Cell culture technology. Cham: Springer International Publishing, 2018, pp.129–137.

[173] Merkhan MM Shephard MT Forsyth NR. Physoxia alters human mesenchymal stem cell secretome. J Tissue Eng 2021; 12: 20417314211056132. 10.1177/20417314211056132 PMC8558798 34733464

[174] Mas-Bargues C Sanz-Ros J Román-Domínguez A , et al. Relevance of oxygen concentration in stem cell culture for regenerative medicine. Int J Mol Sci 2019; 20: 1195. 30857245 10.3390/ijms20051195 PMC6429522

[175] Kwon SY Chun SY Ha YS , et al. Hypoxia enhances cell properties of human mesenchymal stem cells. Tissue Eng Regen Med 2017; 14: 595–604. 30603513 10.1007/s13770-017-0068-8 PMC6171625

[176] Koay EJ Athanasiou KA. Hypoxic chondrogenic differentiation of human embryonic stem cells enhances cartilage protein synthesis and biomechanical functionality. Osteoarthr Cartil 2008; 16: 1450–1456. 10.1016/j.joca.2008.04.007 18541445

[177] Khan WS Adesida AB Hardingham TE. Hypoxic conditions increase hypoxia-inducible transcription factor 2alpha and enhance chondrogenesis in stem cells from the infrapatellar fat pad of osteoarthritis patients. Arthritis Res Ther 2007; 9: R55. 10.1186/ar2211 PMC2206341 17537234

[178] Samal JRK Rangasami VK Samanta S , et al. Discrepancies on the role of oxygen gradient and culture condition on mesenchymal stem cell fate. Adv Healthc Mater 2021; 10: e2002058. 10.1002/adhm.202002058 PMC11469238 33533187

[179] Pezzi A Amorin B Laureano , et al. Effects of hypoxia in long-term in vitro expansion of human bone marrow derived mesenchymal stem cells. J Cell Biochem 2017; 118: 3072–3079. 28240374 10.1002/jcb.25953

[180] Buravkova LB Andreeva ER Gogvadze V , et al. Mesenchymal stem cells and hypoxia: Where are we? Mitochondrion 2014; 19 Pt A: 105–112. 10.1016/j.mito.2014.07.005 25034305

[181] Valorani MG Germani A Otto WR , et al. Hypoxia increases Sca-1/CD44 co-expression in murine mesenchymal stem cells and enhances their adipogenic differentiation potential. Cell Tissue Res 2010; 341: 111–120. 20496083 10.1007/s00441-010-0982-8

[182] Carreau A El Hafny-Rahbi B Matejuk A , et al. Why is the partial oxygen pressure of human tissues a crucial parameter? Small molecules and hypoxia. J Cell Mol Med 2011; 15: 1239–1253. 21251211 10.1111/j.1582-4934.2011.01258.x PMC4373326

[183] Abdollahi H Harris LJ Zhang P , et al. The role of hypoxia in stem cell differentiation and therapeutics. J Surg Res 2011; 165: 112–117. 20080246 10.1016/j.jss.2009.09.057 PMC2891942

[184] Connor DE Paulus JA Dabestani PJ , et al. Therapeutic potential of exosomes in rotator cuff tendon healing. J Bone Miner Metab 2019; 37: 759–767. 31154535 10.1007/s00774-019-01013-z PMC6830879

[185] Shao H Im H Castro CM , et al. New technologies for analysis of extracellular vesicles. Chem Rev 2018; 118: 1917–1950. 29384376 10.1021/acs.chemrev.7b00534 PMC6029891

[186] Camussi G Deregibus M-C Bruno S , et al. Exosome/microvesicle-mediated epigenetic reprogramming of cells. Am j cancer res 2011; 1: 98–110. 21969178 PMC3180104

[187] Abels ER Breakefield XO. Introduction to extracellular vesicles: biogenesis, RNA cargo selection, content, release, and Uptake. Cell Mol Neurobiol 2016; 36: 301–312. 27053351 10.1007/s10571-016-0366-z PMC5546313

[188] Iraci N Leonardi T Gessler F , et al. Focus on extracellular vesicles: physiological role and signalling properties of extracellular membrane vesicles. Int J Mol Sci 2016; 17: 171. 26861302 10.3390/ijms17020171 PMC4783905

[189] Bryzgunova OE Zaripov MM Skvortsova TE , et al. Comparative study of extracellular vesicles from the urine of healthy individuals and prostate cancer patients. PLoS One 2016; 11: e0157566. 10.1371/journal.pone.0157566 PMC4909321 27305142

[190] Tamkovich SN Tutanov OS Laktionov PP. Exosomes: generation, structure, transport, biological activity, and diagnostic application. Biochem (Mosc) Suppl Ser A Membr Cell Biol 2016; 10: 163–173.

[191] Ren K. Exosomes in perspective: a potential surrogate for stem cell therapy. Odontology 2019; 107: 271–284. 30324571 10.1007/s10266-018-0395-9 PMC6465182

[192] Abbaszadeh H Ghorbani F Derakhshani M , et al. Human umbilical cord mesenchymal stem cell-derived extracellular vesicles: a novel therapeutic paradigm. J Cell Physiol 2020; 235: 706–717. 31254289 10.1002/jcp.29004

[193] Spakova T Janockova J Rosocha J. Characterization and therapeutic use of extracellular vesicles derived from platelets. Int J Mol Sci 2021; 22: 9701. 34575865 10.3390/ijms22189701 PMC8468534

[194] Yu H Cheng J Shi W , et al. Bone marrow mesenchymal stem cell-derived exosomes promote tendon regeneration by facilitating the proliferation and migration of endogenous tendon stem/progenitor cells. Acta Biomater 2020; 106: 328–341. 32027991 10.1016/j.actbio.2020.01.051

[195] Li J Liu Z-P Xu C , et al. TGF-β1-containing exosomes derived from bone marrow mesenchymal stem cells promote proliferation, migration and fibrotic activity in rotator cuff tenocytes. Regen Therapy 2020; 15: 70–76. 10.1016/j.reth.2020.07.001 PMC7770343 33426204

[196] Shi Z Wang Q Jiang D. Extracellular vesicles from bone marrow-derived multipotent mesenchymal stromal cells regulate inflammation and enhance tendon healing. J Transl Med 2019; 17: 211. 31238964 10.1186/s12967-019-1960-x PMC6593555

[197] Cui H He Y Chen S , et al. Macrophage-derived miRNA-Containing exosomes induce peritendinous fibrosis after tendon injury through the miR-21-5p/Smad7 Pathway. Mol Ther - Nucleic Acids 2019; 14: 114–130. 30594070 10.1016/j.omtn.2018.11.006 PMC6307349

[198] Li M Jia J Li S , et al. Exosomes derived from tendon stem cells promote cell proliferation and migration through the TGF β signal pathway. Biochem Biophys Res Commun 2021; 536: 88–94. 33370718 10.1016/j.bbrc.2020.12.057

[199] Xu T Xu M Bai J , et al. Tenocyte-derived exosomes induce the tenogenic differentiation of mesenchymal stem cells through TGF-β. Cytotechnology 2019; 71: 57–65. 30599073 10.1007/s10616-018-0264-y PMC6368508

[200] Chisari E Rehak L Khan WS , et al. Tendon healing in presence of chronic low-level inflammation: a systematic review. Br Med Bull 2019; 132: 97–116. 31838495 10.1093/bmb/ldz035

[201] Müller SA Todorov A Heisterbach PE , et al. Tendon healing: an overview of physiology, biology, and pathology of tendon healing and systematic review of state of the art in tendon bioengineering. Knee Surg Sports Traumatol Arthrosc 2015; 23: 2097–2105. 24057354 10.1007/s00167-013-2680-z

[202] Chamberlain CS Clements AEB Kink JA , et al. Extracellular vesicle-educated macrophages promote early Achilles tendon healing. Stem Cells 2019; 37: 652–662. 30720911 10.1002/stem.2988 PMC6850358

[203] Shen H Yoneda S Abu-Amer Y , et al. Stem cell-derived extracellular vesicles attenuate the early inflammatory response after tendon injury and repair. J Orthop Res 2020; 38: 117–127. 31286564 10.1002/jor.24406 PMC6917960

[204] Shi Y Kang X Wang Y , et al. Exosomes derived from bone marrow stromal cells (BMSCs) enhance tendon-bone healing by regulating macrophage polarization. Med Sci Monit 2020; 26: e923328. 10.12659/MSM.923328 PMC7218969 32369458

[205] Shephard MT Merkhan MM Forsyth NR. Human mesenchymal stem cell secretome driven T cell immunomodulation is IL-10 dependent. Int J Mol Sci 2022; 23: 13596. 36362383 10.3390/ijms232113596 PMC9658100

[206] Chen W Huang Y Han J , et al. Immunomodulatory effects of mesenchymal stromal cells-derived exosome. Immunol Res 2016; 64: 831–840. 27115513 10.1007/s12026-016-8798-6

[207] Derwin KA Galatz LM Ratcliffe A , et al. Enthesis repair: challenges and opportunities for effective tendon-to-bone healing. J Bone Joint Surg Am 2018; 100: e109. 10.2106/JBJS.18.00200 PMC6133216 30106830

[208] Wang C Hu Q Song W , et al. Adipose stem cell–derived exosomes decrease fatty infiltration and enhance rotator cuff healing in a rabbit model of chronic tears. Am J Sports Med 2020; 48: 1456–1464. 32272021 10.1177/0363546520908847

[209] Maffulli N Wong J Almekinders LC. Types and epidemiology of tendinopathy. Clin Sports Med 2003; 22: 675–692. 14560540 10.1016/s0278-5919(03)00004-8

[210] Ciardulli MC Lovecchio J Scala P , et al. 3D biomimetic scaffold for growth factor controlled delivery: an in-vitro study of tenogenic events on Wharton’s jelly mesenchymal stem cells. Pharmaceutics 2021; 13: 1448. 34575523 10.3390/pharmaceutics13091448 PMC8465418

[211] Lamparelli EP Lovecchio J Ciardulli MC , et al. Chondrogenic commitment of human bone marrow mesenchymal stem cells in a perfused collagen hydrogel functionalized with hTGF-β1-Releasing PLGA Microcarrier. Pharmaceutics 2021; 13: 399. 33802877 10.3390/pharmaceutics13030399 PMC8002618

[212] Scala P Lovecchio J Lamparelli EP , et al. Myogenic commitment of human stem cells by myoblasts Co-culture: a static vs. A dynamic approach. Artif Cells Nanomed Biotechnol 2022; 50: 49–58. 35188030 10.1080/21691401.2022.2039684

[213] Webb WR Dale TP Lomas AJ , et al. The application of poly(3-hydroxybutyrate-co-3-hydroxyhexanoate) scaffolds for tendon repair in the rat model. Biomaterials 2013; 34: 6683–6694. 23768899 10.1016/j.biomaterials.2013.05.041

[214] Lyu K Liu T Chen Y , et al. A “cell-free treatment” for tendon injuries: adipose stem cell-derived exosomes. Eur J Med Res 2022; 27: 75. 35643543 10.1186/s40001-022-00707-x PMC9148514

[215] Zhang M Liu H Cui Q , et al. Tendon stem cell-derived exosomes regulate inflammation and promote the high-quality healing of injured tendon. Stem Cell Res Ther 2020; 11: 402. 32943109 10.1186/s13287-020-01918-x PMC7499865

