# Corrigendum to “Tendon tissue engineering: An overview of biologics to promote tendon healing and repair”

**DOI:** 10.1177/20417314251380714

**Published:** 2025-09-25

Citro V, Clerici M, Boccaccini AR, Della Porta G, Maffulli N, Forsyth NR. Tendon tissue engineering: An overview of biologics to promote tendon healing and repair. *J. Tissue Eng*. 2023;14. doi:10.1177/20417314231196275.

The authors failed, through clerical error, to include the citation of “Hedgehog signaling underlying tendon and enthesis development and pathology” by Fang et al^21^. in Figure 3.

The authors have provided an updated figure legend with information related to the license provided by another publisher.

The authors apologize for this inadvertent error.

**Figure 3.** Expression of tendon markers in tenocytes during tendon development. (A) Mesenchymal cells differentiate into Scx-expressing tendon progenitor cells, which also partially express Sox9. Scx+Sox9+ progenitor cells differentiate into the tenocytes which are located near the bone in the enthesis. (B) In mouse limbs, Scx expression begins to increase at E9.5 and continues to increase until tenocyte maturation. Slight Mkx expression is detectable in the tendon at E12.5, after the emergence of Scx and robust Mkx mRNA expression at E13.5 and E14.5, stages at which the tendon progenitors undergo condensation and differentiation. Egr1 transcripts are first expressed at E12.5 in Scx domains forming tendons, and then they are expressed in long tendons at E16.5. Egr2 is first detectable in E14.5 limb tendons and is generally expressed in all limb tendons by E16.5. Tnmd is highly expressed in E14.5 and is considered a late tendon marker. Adapted from He et al.^20^, 2022, reproduced under a Creative Commons CC-BY license, and from Fang et al.^21^, 2022, with permission from Elsevier Science.

**Reference**

21. Fang F, Sup M, Luzzi A, et al. Hedgehog signaling underlying tendon and enthesis development and pathology. *Matrix Biol* 2022; 105: 87–103.

